# An immunotherapy response prediction model derived from proliferative CD4^+^ T cells and antigen-presenting monocytes in ccRCC

**DOI:** 10.3389/fimmu.2022.972227

**Published:** 2022-08-25

**Authors:** Kun Zheng, Lianchong Gao, Jie Hao, Xin Zou, Xiaoyong Hu

**Affiliations:** ^1^ Department of Urology, Shanghai Jiao Tong University Affiliated Sixth People’s Hospital, Shanghai, China; ^2^ Yantai Institute, China Agricultural University, Yantai, China; ^3^ Institute of Clinical Science, Zhongshan Hospital, Fudan University, Shanghai, China; ^4^ Center for Tumor Diagnosis & Therapy, Jinshan Hospital, Fudan University, Shanghai, China; ^5^ Department of Pathology, Jinshan Hospital, Fudan University, Shanghai, China

**Keywords:** clear cell renal cell carcinoma (ccRCC), immune checkpoint blockade (ICB) therapy, response prediction model, single-cell RNA-seq, machine learning (ML)

## Abstract

Most patients with clear cell renal cell carcinoma (ccRCC) have an impaired response to immune checkpoint blockade (ICB) therapy. Few biomarkers can predict responsiveness, and there is insufficient evidence to extend them to ccRCC clinical use. To explore subtypes and signatures of immunocytes with good predictive performance for ICB outcomes in the ccRCC context, we reanalyzed two ccRCC single-cell RNA sequencing (scRNA-seq) datasets from patients receiving ICB treatment. A subtype of proliferative CD4^+^ T cells and regulatory T cells and a subtype of antigen-presenting monocytes that have good predictive capability and are correlated with ICB outcomes were identified. These findings were corroborated in independent ccRCC ICB pretreatment bulk RNA-seq datasets. By incorporating the cluster-specific marker genes of these three immunocyte subtypes, we developed a prediction model, which reached an AUC of 93% for the CheckMate cohort (172 samples). Our study shows that the ICB response prediction model can serve as a valuable clinical decision-making tool for guiding ICB treatment of ccRCC patients.

## Introduction

Clear cell renal cell carcinoma (ccRCC) is the most common histological subtype and accounts for more than 70% of all renal cell carcinoma (RCC) (1). The global incidence has shown an upward trend in recent years (2). Large-scale tumor transcriptome analysis revealed that ccRCC was highly infiltrated by immune cells (3), and most notably, its T-cell infiltration was the highest among tumor types within The Cancer Genome Atlas (TCGA) (3). Based on this feature, immune checkpoint blockade (ICB) therapy has significantly improved advanced ccRCC treatment (4, 5). However, a large proportion of ccRCC patients do not respond to this therapy for unclear reasons (6). Such diversity in response to ICB highlights the necessity of identifying predictive biomarkers.

Previous whole-exome sequencing (WES) and transcriptome sequencing of tumors identified several factors associated with ICB outcomes, such as high T/low myeloid cell infiltration (7), high B-cell (8)/CD4 memory T cell abundance (9), elevated PD-L1 expression (10), high tumor mutational burden (TMB) (11), and high similarity/diversity of TCR clonality (12, 13), which are associated with ICB responses. Exhausted/dysfunctional CD8^+^ T cells (13, 14), anti-inflammatory/M2-like tumor-associated macrophages (TAMs) (13, 14), defects in IFNγ signaling (15) or antigen processing and presentation (16) all contribute to resistance. However, ccRCC is unique compared to other tumors in response to immunotherapy. For example, CD8^+^ T-cell infiltration (17), tumor mutation burden (11), frameshift insertions and deletions (fsINDELs) (18), and HLA heterozygosity (19) have been described as pro-response effects in other tumor types, whereas none of them have been shown to be associated with ICB response in ccRCC (4, 7, 20). To date, the factors driving ICB resistance remain largely unknown. Effective biomarkers for predicting the ccRCC ICB response in clinical practice are still lacking.

In this study, we reanalyzed two independent ccRCC single-cell RNA sequencing (scRNA-seq) datasets (12, 14) to construct a prediction model. We found that proliferative CD4^+^ T cells (MKI67^+^ CD4Ts) and proliferative regulatory T cells (MKI67^+^ Tregs) can promote ICB resistance, and a subset of antigen-presenting monocytes is related to the ICB response. Based on these findings, we developed an effective ICB response signature, and this signature was validated in several ICB pretreatment bulk RNA-seq datasets. Overall, this signature reached an AUC of 93% for a 172-sample cohort and is more effective than previously published ICB response signatures. These findings extend our understanding of factors associated with the ICB response and provide a potentially powerful response prediction model for ICB clinical treatment.

## Results

### CD4^+^ T, Treg, and monocyte cells are associated with the ICB response/resistance in ccRCC

We reanalyzed two independent publicly available ccRCC scRNA-seq datasets [Bi’s dataset (14) and Au’s dataset (12)], which were both from ccRCC patients treated with PD-1/PD-L1 monoclonal antibodies. Then, the observations from single-cell datasets were validated across multiple ccRCC bulk RNA-seq datasets.

Details of all scRNA-seq and bulk RNA-seq datasets used in this study are described in the Methods, and the metadata for all samples are available in **Supplementary Table 1**. Patients were divided into responders (R), including complete response (CR) and partial response (PR), and nonresponders (NR), including stable disease (SD) and progressive disease (PD), according to the response evaluation criteria in solid tumors (RECIST) (21). Not evaluable (NE) samples from all cohorts were omitted from the RECIST response analysis but remained from the survival analysis.

**Figure 1:**
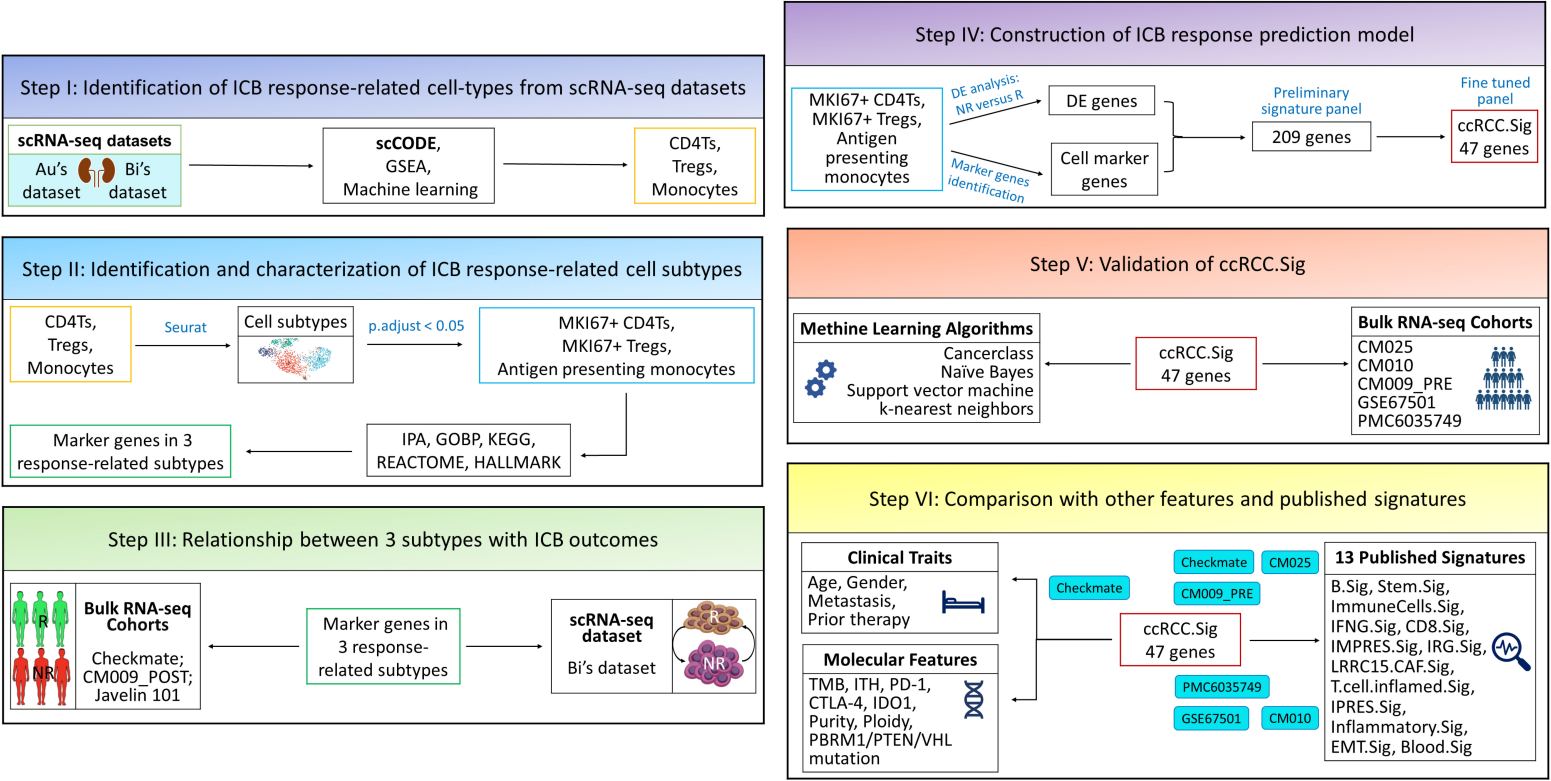
The overall design of this study. Au and Bi are scRNA-seq datasets, and CheckMate, CM025, CM010, CM009, Javelin 101, GSE67501 and PMC6035749 are bulk RNA-seq datasets. GSEA, gene set enrichment analysis; DE, differentially expressed; ICB, immune checkpoint blockade; IPA, ingenuity pathway analysis.

The overall design of this study is summarized in **Figure 1**. We used the cell types of Bi’s dataset (n.patients = 7, R = 2, NR = 2, NoICB = 3) and Au’s dataset (n.patients = 2, R = 1, NR = 1) defined by the original studies (**Figure S1A**). There were ten cell types from Bi’s dataset, i.e., B cells, CD4^+^ T cells (CD4Ts), CD8^+^ T cells (CD8Ts), dendritic cells (DCs), monocytes, tumor-associated macrophages (TAMs), natural killer cells (NKs), natural killer T cells (NKTs), regulatory T cells (Tregs) and cancer cells, as well as three cell types from Au’s dataset: CD4Ts, CD8Ts and Tregs. **Figure S1B** shows the visualization results of the 12 immune cell types (excluding cancer cells) in two-dimensional space *via* uniform manifold approximation and projection (UMAP).

According to the workflow in **Figure S1A**, we identified the differentially expressed (DE) genes of R and NR in the 13 cell types using the scCODE R package (22) and obtained a total of 26 DE gene lists (Extended data 1, see *Study Design* for details). We sought to identify gene sets that were effective predictors of ICB outcomes. From the Molecular Signatures Database (MSigDB) (23), the 26 DE gene lists were found to be enriched in 1008 gene sets, including GOBP, Hallmark, KEGG, and Reactome (Extended data 2, see *Study Design* for details). The predictive capabilities of the 1008 gene sets were evaluated in terms of receiver operating characteristic (ROC) curves obtained with the Cancerclass R package (24) in the CheckMate cohort (20) (n.patients = 172, R = 39, NR = 133, see *Methods* for details). ROC p values for these 1008 gene sets were visualized and are listed in **Figures S2 A–D** and Extended data 2.

We found that the GOBP gene sets had overall better predictive performance than Hallmark, KEGG and Reactome. To identify the cell types with good predictive capability, we examined the enriched GOBP gene sets with ROC p values < 0.05 (without correction) within each DE gene list. A DE gene list was considered significant if no less than half of its enriched gene sets had ROC p values < 0.05. Next, based on the same criteria, significant DE gene lists were identified in Hallmark, KEGG, and Reactome. As a result, we identified 7 prediction-related DE gene lists (**Figure S2A**). The DE gene lists were further reduced to 3 with FDR corrected ROC p values (**Figure S2E**): the monocytes from Bi’s dataset responders (Bi’s_Mono.R), and CD4Ts and Tregs from Au’s dataset nonresponders (Au’s_CD4T.NR and Au’s_Treg.NR). We speculated that the CD4T, Treg and monocyte subtypes might be correlated with ICB responses.

### MKI67^+^ CD4Ts and MKI67^+^ Tregs were enriched in nonresponders

**Figure 2:**
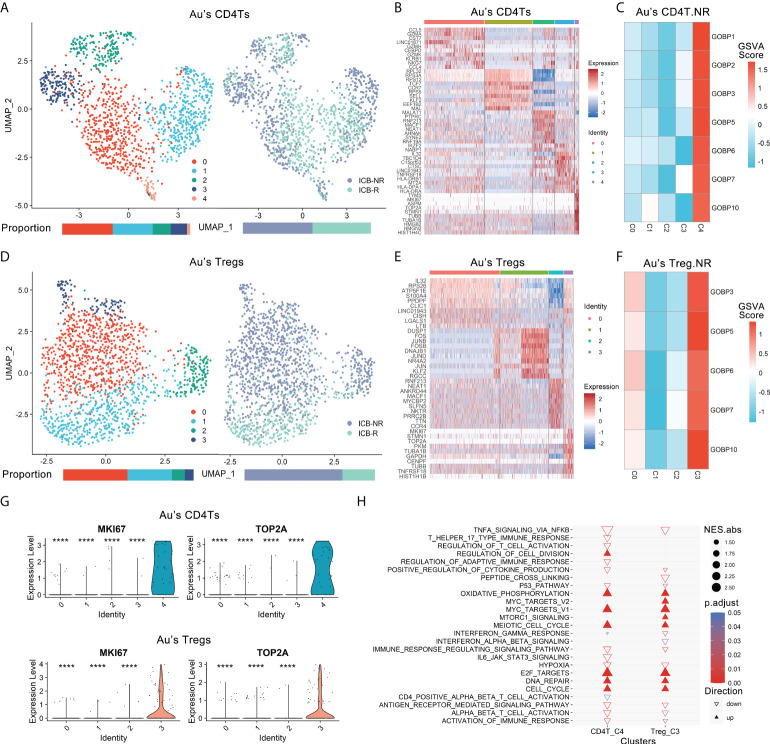
Proliferative subtypes of CD4Ts and Tregs were enriched in nonresponders. Au’s CD4Ts and Tregs scRNA-seq datasets were analyzed. **(A)** UMAP plot of Au’s dataset’s CD4Ts that were classified into 5 subclusters from R and NR of distinct ICB outcomes. Bar plots show cell proportions grouped by clusters (left) and ICB outcomes (right). **(B)** Heatmap of scaled normalized expression for the top 10 specific marker genes of Au’s CD4T subclusters as identified by a two-sided Wilcoxon rank-sum test with FDR correction (q < 0.05). **(C)** ICB response prediction-related CD4T subcluster was identified by locating the effective (ROC p.adjust < 0.05) predictive gene set expression *via* gene set variation analysis (GSVA). **(D)** UMAP plot of Au’s Treg dataset that was classified into 4 subclusters of distinct immune checkpoint therapy outcomes from R and NR. Bar plots show cell proportions grouped by clusters (left) and ICB outcomes (right). **(E)** Heatmap of scaled normalized expression for the top 10 specific marker genes of Au’s Treg subclusters as identified by a two-sided Wilcoxon rank-sum test with FDR correction. **(F)** ICB response prediction-related Treg subtype was identified by locating the effective predictive gene set expression *via* GSVA. **(G)** Violin plot of the expression levels of proliferative marker genes in Au’s CD4Ts and Treg subclusters. A two-sided Wilcoxon test was used to determine significance between the subclusters of interest and others. ****P < 0.0001. **(H)** GOBP, Hallmark, KEGG and Reactome analysis results of Au’s CD4Ts subcluster 4 (CD4T_C4) and Au’s Treg subcluster 3 (Treg_C3) compared with other subclusters.

The two significant nonresponder-associated DE gene lists: Au’s_CD4T.NR and Au’s_Treg.NR in Au’s dataset was further analyzed. The 1308 CD4Ts (NR.cells = 712, R.cells = 596) and 1501 Tregs (NR.cells = 126, R.cells = 375) were clustered into five CD4Ts subclusters and four Tregs subclusters by Seurat (25), respectively (**Figures 2A, D**). Subsequently, cluster-specific marker genes were identified by FindAllMarkers (Seurat), and the expression heatmap of the top 10 marker genes is shown in **Figures 2B, E**. The classical marker genes of CD4Ts and Tregs were highly expressed in all subclusters (**Figures S3A, S3B**).

There were 7 gene sets of Au’s_CD4T.NR and 5 gene sets of Au’s_Treg.NR with ROC p.adjust < 0.05 (**Figure S2E**). We analyzed the expression of these gene sets *via* gene set variation analysis (GSVA) (26) and found that they were highly expressed in CD4T subcluster 4 (CD4T_C4, NR.cells = 24, R.cells = 8) (**Figure 2C**, **Figure S3C**) and Treg subcluster 3 (Treg_C3, NR.cells = 96, R.cells = 5) (**Figure 2F**, **Figure S3D**). Therefore, both CD4T_C4 and Treg_C3 cells were likely associated with ICB resistance.

Further analysis of these two subclusters revealed that although they belong to different cell types, they are both proliferative cell populations. Specifically, they both highly expressed MKI67, TOP2A, TUBB, TUBA1B, STMN1, TYMS and other proliferation markers (27, 28) (**Figure 2G**, **Figures S3E, S3F**). The GOBP, Hallmark, KEGG and Reactome analyses of their marker genes showed that cell cycle-related pathways and metabolic activities (e.g., DNA repair and oxidative phosphorylation [OXPHOS], etc.), oncogenic pathways such as MYC (29) and E2F (30) targets were dramatically enriched (**Figure 2H**, Extended data 3). In contrast, pathways associated with tumor suppression, such as immune activation-related pathways (31), antigen presentation and processing (16), IFNγ responses (15, 32), TNFα signaling (33), and P53 pathways (34), were remarkably suppressed in both subclusters (**Figure 2H**, Extended data 3). In addition, CD4T_C4 also downregulated anticancer immune responses and cytokine-related pathways (35), while Treg_C3 downregulated anticancer IFNα and IFNβ signaling (15, 32) and activated oncogenic mTORC1 signaling (36) (**Figure 2H**, Extended data 3). Their top 20 pathways sorted by the absolute value of the normalized enrichment score (NES) also confirmed the above observation (**Figures S3G, S3H**). Next, we used ingenuity pathway analysis (IPA) for further verification. IPA showed that CD4T_C4 and Treg_C3 cells have significantly activated cell cycle-related kinetochore, chromosomal replication, cyclins and mitosis, as well as oxidative phosphorylation, while cell cycle-related checkpoints were inhibited in CD4T_C4 cells (**Figures S3I, S3J**). Such observations were consistent with previous studies, i.e., dysfunction of cell cycle-related checkpoints often results in genomic instability and oncogenesis (37). Therefore, CD4T_C4 and Treg_C3 cells are proliferative subtypes and may impair antitumor immunotherapy. In view of the high expression of proliferation markers and enrichment of cell cycle-related pathways in both CD4T_C4 and Treg_C3, we annotated them as MKI67^+^ CD4Ts and MKI67^+^ Tregs, respectively.

### Validation of MKI67^+^ CD4Ts and MKI67^+^ Treg signatures in independent datasets

As mentioned above, we preliminarily confirmed that MKI67^+^ CD4Ts and MKI67^+^ Tregs were related to ICB resistance. To further validate this finding, we investigated whether tumors enriched with these two cell subtypes were more susceptible to ICB resistance.

**Figure 3:**
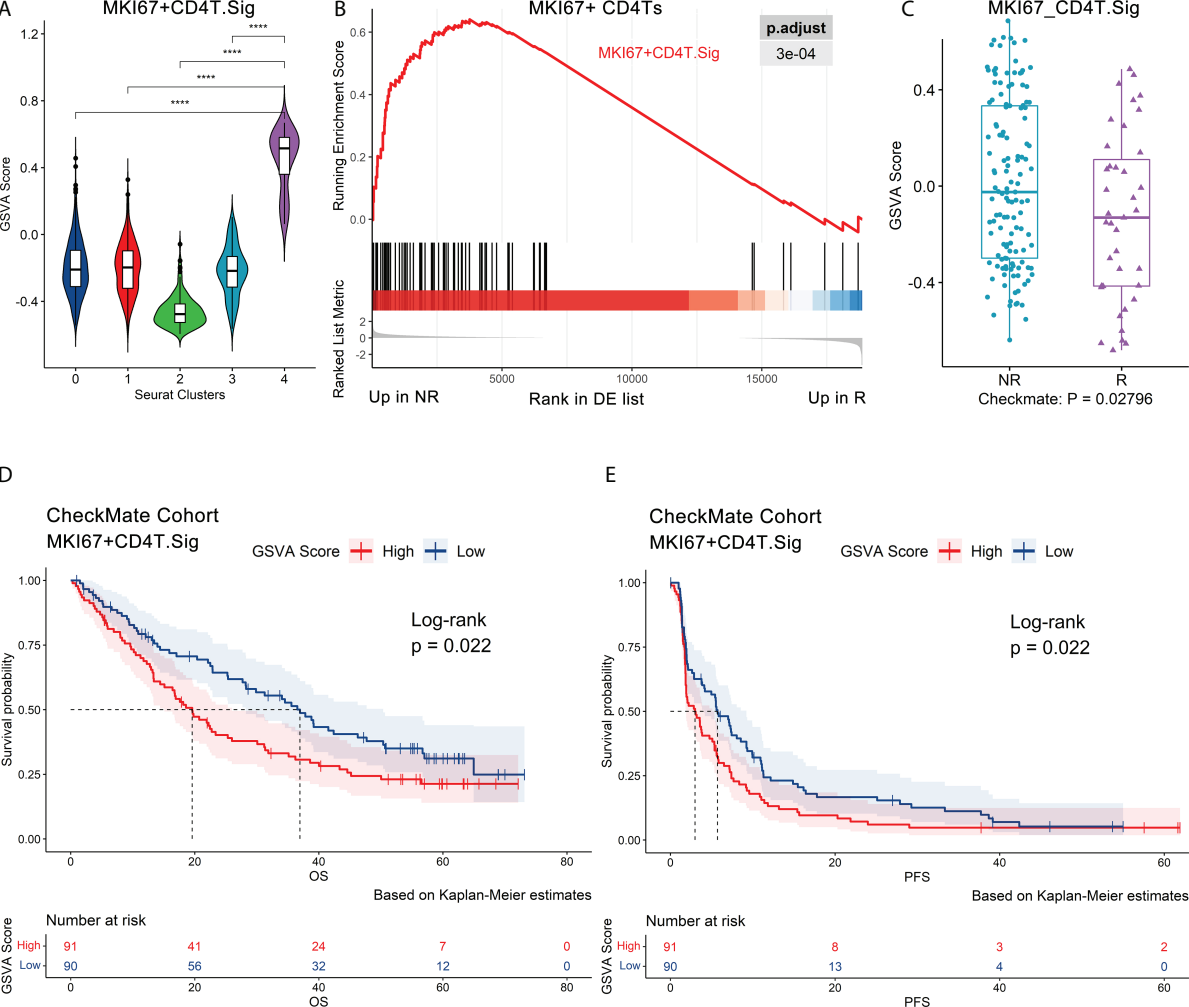
Validation of the MKI67^+^ CD4Ts signature using independent bulk RNA-seq datasets. Au’s CD4Ts scRNA-seq dataset and CheckMate cohort were analyzed. **(A)** Violin plot of a 70-gene signature (MKI67^+^CD4T. Sig) GSVA scores showed that it can specifically characterize proliferative subcluster 4 of CD4Ts (MKI67^+^ CD4Ts). Centerline, median. Box limits, upper and lower quartiles. Whiskers, 1.5 interquartile range. Points beyond whiskers, outliers. A two-sided Wilcoxon test was used to determine significance. ****P < 0.0001. **(B)** GSEA revealed that MKI67^+^CD4T.Sig was enriched in NR of MKI67^+^ CD4Ts. The p value was FDR-adjusted by the FDR method. **(C)** Boxplot validated that MKI67^+^CD4T. The Sig GSVA scores of NR were significantly higher than those of R in the CheckMate cohort (n.patients = 172, R = 39, NR = 133) *via* GSVA analysis. Centerline, median. Box limits, upper and lower quartiles. Whiskers, 1.5 interquartile range. Points beyond whiskers, outliers. A two-sided Wilcoxon test was used to determine significance. **(D), (E)** Survival analysis was performed on GSVA scores for MKI67^+^CD4T cells. Sig in the pretreatment CheckMate cohort (n.patients = 181, R = 39, NR = 133, NE = 9, NEs were not removed for survival analysis) based on the Kaplan–Meier method. The groups were dichotomized at the median GSVA score, and the log-rank test was used to determine significance. Dashed line: median survival time. Color range: 95% confidence interval (CI).

The 70 marker genes of MKI67^+^ CD4Ts (annotated as MKI67^+^CD4T.Sig) were characterized by cell cycle, MYC, E2F targets and OXPHOS (**Figure S4A**, Extended data 4). The GSVA (26) score of MKI67^+^CD4T.Sig was significantly higher than the other CD4Ts subclusters (**Figure 3A**, **Figure S4B**). These genes were enriched in nonresponders (**Figure 3B**), and the GSVA score was notably higher in nonresponders as well (**Figure S4C**). Subsequently, we validated MKI67^+^CD4T.Sig in the pretreatment CheckMate cohort (n.patients = 172, R = 39, NR = 133) (20) and found that nonresponders had higher GSVA scores (p = 0.028) (**Figure 3C**). Consistently, gene set enrichment analysis (GSEA) showed significant enrichment of MKI67^+^ CD4T.Sig in nonresponders compared to responders (**Figure S4D**).

Next, we analyzed the impact of MKI67^+^ CD4Ts on the prognosis of ICB treatment patients. It was found that in the pretreatment CheckMate cohort (n.patients = 181, R = 39, NR = 133, NE = 9, NEs were not removed for survival analysis, the same below), the group with low MKI67^+^CD4T.Sig GSVA scores were associated with significantly higher overall survival (OS, p = 0.022) and progression-free survival (PFS, p = 0.022) (**Figures 3D, E**). Subsequently, we corroborated this finding in the Javelin101 cohort (4) with 726 pretreatment bulk RNA-seq data (see Methods for details). Consistent results were obtained, i.e., the group with low GSVA scores had markedly longer PFS (p < 0.0001) (**Figure S4E**). To eliminate the effects of sunitinib treatment on the above results, we extracted and analyzed the avelumab plus axitinib group (4) bulk RNA-seq data (n.patients = 354), and the results were the same (p = 0.022, **Figure S4F**). In addition, we validated the results in the posttreatment bulk RNA-seq dataset of the CheckMate 009 (CM009) cohort (38) (CM009_POST, n.patients = 55, R = 5, NR = 47, NE = 3, see Methods for details). Lower MKI67^+^CD4T.Sig GSVA scores after ICB treatment were also significantly correlated with prolonged OS (p = 0.018) and PFS (p = 0.0093) (**Figures S4G, S4H**). Finally, univariate logistic regression with MKI67^+^CD4T.Sig in predicting ICB outcomes obtained an AUC of 0.62 (p = 0.008, **Figure S4I**).

Similar procedures were used to verify MKI67^+^ Tregs. The MKI67^+^Treg.Sig contained 80 genes involved in the cell cycle, MYC and E2F targets, mTORC1 signaling, OXPHOS and DNA repair (**Figure S5A**, Extended data 4). Violin and feature plots of GSVA scores showed that this gene set had distinguished specificity for characterizing MKI67^+^ Tregs **(** **Figures S5B, S5C**). In both single-cell and bulk RNA-seq datasets, nonresponders had higher MKI67^+^Treg.Sig GSVA scores than responders (p values 0.046 and 0.0047, respectively) (**Figures S5D, S5E**) and had significant MKI67^+^Treg.Sig enrichment (p < 0.001 and 0.0013, respectively) (**Figures S5F, S5G**). Survival analysis of the CheckMate, Javelin 101 and CM009_POST cohorts consistently demonstrated that high levels of MKI67^+^ Tregs, either pre- or post-ICB treatment, were all associated with worse OS and PFS (**Figures S5H–S5L**). The above results confirmed that MKI67^+^ CD4Ts and MKI67^+^ Tregs could shorten OS and PFS and promote ICB resistance. Similarly, univariate logistic regression showed that MKI67^+^Treg.Sig performed well in predicting ICB outcomes (AUC = 0.64, p = 0.011, **Figure S5M**).

Finally, we used an independent single-cell dataset, Bi’s dataset, to validate the universality of MKI67^+^ CD4Ts and MKI67^+^ Tregs characteristics. The 2245 CD4Ts (NR.cells = 818, R.cells = 641, NoICB.cells = 786) and 740 Tregs (NR.cells = 89, R.cells = 307, NoICB.cells = 344) from Bi’s dataset were clustered into five CD4Ts subclusters (**Figure S6A**) and three Tregs subclusters (**Figure S6D**), respectively. We matched subclusters with high levels of MKI67^+^ CD4Ts and MKI67^+^ Treg signatures through GSVA with CD4T_C0 (NR.cells = 56, R.cells = 347, NoICB.cells = 277) and Treg_C0 (NR.cells = 20, R.cells = 156, NoICB.cells = 106) (**Figures S6B, S6E**), respectively. As previously described, there were 7 gene sets of Au’s_CD4T.NR and 5 gene sets of Au’s_Treg.NR with ROC p.adjust < 0.05 (**Figure S2E**). In parallel, we found that these gene sets were also specifically enriched in CD4T_C0 and Treg_C0 of Bi’s dataset (**Figures S6C, S6F**), respectively. Such consistency was also confirmed by overall expression (OE) (39). The results showed that nonresponders in CD4T_C0 had higher MKI67^+^ CD4Ts-OE signals than responders (p = 0.018, **Figure S6G**), and a similar result was observed in Treg_C0 cells (p = 0.0004, **Figure S6H**).

### Antigen-presenting monocytes are associated with the ICB response

**Figure 4:**
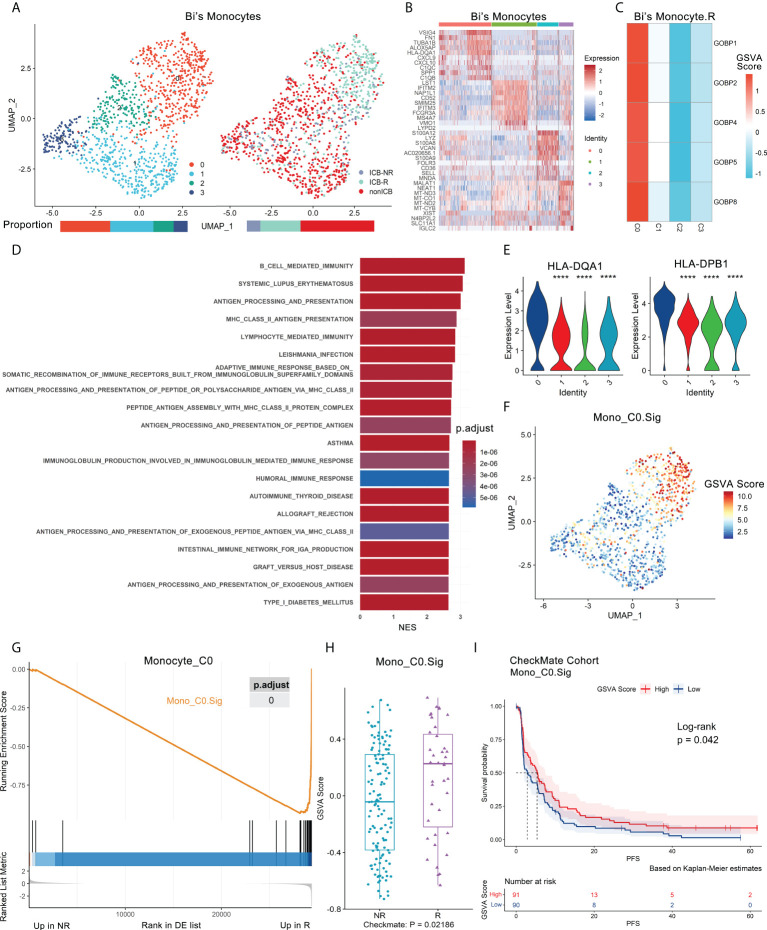
Antigen-presenting monocyte subtype promotes ICB response and is validated in independent bulk RNA-seq datasets. Bi’s monocyte scRNA-seq dataset and the CheckMate cohort were analyzed. **(A)** UMAP plot of monocytes from Bi’s dataset that were classified into 4 subclusters from ICB-R, ICB-NR and NoICB samples. Bar plots show cell proportions grouped by clusters (left) and ICB outcomes (right). **(B)** Heatmap of scaled normalized expression for the top 10 specific marker genes of Bi monocyte subclusters as identified by a two-sided Wilcoxon rank-sum test with FDR correction (q < 0.05). **(C)** The ICB response prediction-related monocyte subcluster was identified by locating the effective predictive gene set expression *via* GSVA. **(D)** GOBP, Hallmark, KEGG and Reactome analysis results of Bi’s monocyte subcluster 0 compared with other subclusters. The top 20 pathways sorted by the absolute value of the normalized enrichment score (NES) are listed. **(E)** Violin plot of the expression levels of MHC class II genes in Bi’s monocyte subclusters. A two-sided Wilcoxon test was used to determine significance between the subclusters of interest and others. ****P < 0.0001. **(F)** Feature plot of a 45-gene signature (Mono_C0. Sig) GSVA scores showed that it can specifically characterize the antigen-presenting subcluster 0 of monocytes (Mono_C0). **(G)** GSEA showed that Mono_C0. Sig was enriched in R of Mono_C0. The p value was FDR-adjusted by the Benjamini–Hochberg method. **(H)** Boxplot validated that Mono_C0. The Sig GSVA scores of R were significantly higher than those of NR in the CheckMate cohort (n.patients = 172, R = 39, NR = 133) *via* GSVA analysis. Centerline, median. Box limits, upper and lower quartiles. Whiskers, 1.5 interquartile range. Points beyond whiskers, outliers. A two-sided Wilcoxon test was used to determine significance. **(I)** Kaplan–Meier plot of progression-free survival (PFS) data for the pretreatment CheckMate cohort (n.patients = 181, R = 39, NR = 133, NE = 9) on the basis of Mono_C0. Sig GSVA scores. The groups were dichotomized at the median GSVA score, and the log-rank test was used to determine significance. Dashed line: median survival time. Color range: 95% CI.

As mentioned before, some gene sets enriched in responder monocytes (Bi’s_Mono.R) had good predictive capability (**Figure S2E**, Extended data 2), which suggested that certain subtypes of monocytes might be associated with the ICB response. The UMAP of monocytes (NR.cells = 122, R.cells = 364, NoICB.cells = 669) in Bi’s dataset showed four subclusters (**Figures 4A, B**). The canonical marker genes of monocytes were highly expressed in all subclusters (**Figure S7A**).

There were five gene sets with ROC p.adjust < 0.05 in Bi’s_Mono.R (**Figure S2E**), and they were uniquely highly expressed in subcluster 0 (Mono_C0, NR.cells = 56, R.cells = 262, NoICB.cells = 134) (**Figure 4C**, **Figure S7B**). Mono_C0 highly expressed MHC class II antigen and IFNγ response-related genes, including HLA-DRB1, HLA-DPA1, HLA-DPB1, CD74 and TUBA1B (**Figures 4B, E**). GSEA showed that Mono_C0 promoted the ICB response through multiple pathways, including antigen processing and presentation (16), adaptive immune response (31) and IFNγ signaling pathways (40) (**Figure 4D**, Extended data 3). In addition, IPA showed significant inhibition of the PD-1/PD-L1 pathway (**Figure S7C**), indicating that Mono_C0 may play an important role in immunotherapy.

To validate the role of antigen-presenting monocytes in immunotherapy, a Mono_C0.Sig gene panel was constructed based on its marker genes. This gene panel consisted of 45 genes, all of which represented characteristic pathways such as antigen processing and presentation, IFNγ signaling, and immune response **(** **Figure S7D**, Extended data 4), with good specificity for characterizing Mono_C0 (**Figure 4F**, **Figure S7E**) and significant enrichment in responders (**Figure 4G**, **Figure S7F**). We then validated Mono_C0.Sig in the CheckMate cohort (n.patients = 172). GSVA and GSEA analyses revealed significant enrichment in responders (**Figure 4H**, **Figure S7G**). Survival analysis showed that higher GSVA scores indicated better PFS (p = 0.042) (**Figure 4I**), although the GSVA scores were not correlated with OS (p = 0.95) (**Figure S7H**). Consistently, the same results were observed in the CM009_POST cohort (**Figures S7I, S7J**). The results suggested that ccRCC patients with abundant Mono_C0 subtypes had prolonged PFS after ICB treatment. Such observations were consistent with previous reports, e.g., MHC class II molecule expression was related to the ICB response and prognosis improvement (41, 42). Furthermore, univariate logistic regression analysis showed that Mono_C0.Sig could predict ICB outcomes with an AUC of 0.63 (p = 0.019, **Figure S7K**).

Comparing the NoICB and ICB treatment samples from Bi’s dataset, the expression of Mono_C0. Sig was elevated after ICB treatment (p < 0.001) (**Figure S7L**). We further analyzed a subdataset from the CM009 cohort (CM009_Paired), which contained paired kidney biopsy samples of ICB pretreatment and week 4 treatment from 42 patients (38). A similar dynamic pattern was observed (Wilcoxon’s paired test p = 0.0068) (**Figure S7M**). Evaluation in the CM009_POST cohort indicated the upregulation of Mono_C0.Sig was more obvious in ICB responders than nonresponders (p = 0.038) (**Figure S7N**).

### ICB response prediction model construction and validation

As MKI67^+^ CD4Ts and MKI67^+^ Tregs tended to impair immunotherapy efficacy and antigen-presenting monocytes promoted the ICB response, we compared the predictive capability of these three cell subtypes with that of other CD4Ts, Tregs and monocytes subclusters. Specifically, the top 500 genes sorted in ascending order of adjusted p value (obtained by FindAllMarkers of Seurat) were selected to identify the top 10 enriched GOBP gene sets for each cell subcluster of CD4Ts, Tregs and monocytes (Extended data 5, **Figure S8**). We subsequently tested the predictive capability of each individual gene set in the CheckMate cohort (n.patients = 172) using Cancerclass. Most gene sets associated with MKI67^+^ CD4Ts, MKI67^+^ Tregs and antigen-presenting monocytes had high predictive capability compared with the rest (**Figure S9A-S9C**, Extended data 5). This provided a foundation to develop a signature panel for ICB outcome prediction based on the three cell subtypes.

**Figure 5:**
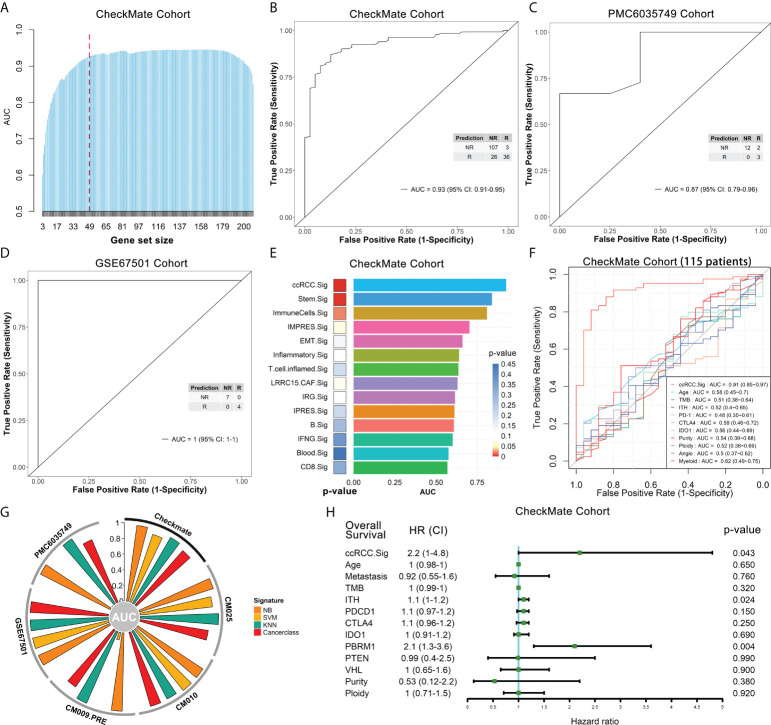
The ccRCC.Sig signature can effectively predict ICB outcomes in ccRCC patients. The bulk RNA-seq datasets CheckMate (n = 172, R = 39, NR = 133), CM025 (n = 111, R = 25, NR = 86), CM010 (n = 45, R = 11, NR = 34), CM009_PRE (n = 56, R = 9, NR = 47), PMC6035749 (n = 17, R = 5, NR = 12) and GSE67501 (n = 11, R = 4, NR = 7) were analyzed. **(A)** Bar graph showing the AUC of gene combinations with maximal AUC for each cycle (different gene-number combinations). Dotted line: 47-gene combination - ccRCC.Sig. **(B)** ccRCC.Sig had significantly high predictive value for ICB outcomes in the CheckMate cohort. **(C), (D)** ccRCC.Sig accurately predicted the ICB outcomes in the validation **(C)** PMC6035749 and **(D)** GSE67501 cohorts. **(E)** Comparison of the performance (AUC and p value) of ccRCC.Sig with 13 other ICB response signatures. **(F)** Comparison of the performance (AUC) of ccRCC.Sig with other clinical traits and molecular features in the CheckMate cohort (115 samples remained after deleting missing data samples). **(G)** Validation of ccRCC.Sig using three other machine learning algorithms. SVM, support vector machine; NB, naïve Bayes; KNN, k-nearest neighbors. **(H)** Univariate Cox regression analyses of ccRCC.Sig, clinical traits and molecular features. TMB, tumor mutational burden; ITH, intratumor heterogeneity.

Based on the selected gene sets with significant predictive capability (p.adjust < 0.05) (**Figures S9A-S9C**), we developed the overall gene signatures using Cancerclass according to the workflow shown in **Figure S8** (see *Methods* for details). Initially, 209 genes were obtained (**Figure S8**, orange box), and GSEA showed that the top 20 pathways were mainly the cell cycle, DNA metabolism and repair, E2F and MYC targets (**Figure S10A**). Most of these pathways contributed to the poor immune response according to previous studies (29, 30, 43). The top 10 pathways were used to predict the outcomes of the CheckMate cohort, and these pathways had good prediction performance, with AUCs between 0.68 and 0.83 (**Figure S10B**). To construct a more effective prediction model, we executed the cycle algorithm shown in **Figure S8** (purple box, see *Methods* for details) and examined the AUCs for combinations with different numbers of genes (**Figure 5A**, Extended data 6). A good prediction model should balance the highest reliability and fewest genes. Therefore, to balance these two factors, we selected the 47-gene combination as our ccRCC ICB response prediction signature - ccRCC.Sig in downstream analysis (**Figure 5A**, dotted line). Details of these 47 genes can be found in **Supplementary Table 2**. The ccRCC. Sig could accurately discriminate between responders and nonresponders in the CheckMate cohort (n.patients = 172, R = 39, NR = 133). Specifically, the AUC of this signature was 0.93 (95% confidence interval [CI]: 0.91-0.95), the sensitivity was 80% (74-86%), the specificity was 92% (81-98%), and p = 7.2e-07 (**Figure 5B**). This response prediction signature gathers characteristic genes from MKI67^+^ CD4Ts, MKI67^+^ Treg cells and antigen-presenting monocyte subtypes (**Figures S11A–C**). In addition, we tested the performance of 30-gene and 20-gene combinations in the CheckMate cohort. However, their predictive performances were less than that of ccRCC.Sig, they were also effective, with AUCs of 0.89 (0.87-0.91) and 0.86 (0.84-0.89), respectively (**Figures S12A, B**).

Subsequently, we validated the performance of ccRCC.Sig in independent pretreatment bulk RNA-seq datasets. Because the CheckMate cohort (20) is integrated data of the CheckMate 025 (CM025) (5), CheckMate 010 (CM010) (44), and CM009 (45) cohorts (see Methods for details), we first validated separately in each of these three cohorts. For the CM025 (n.patients = 111, R = 25, NR = 86) and CM010 cohorts (n.patients = 45, R = 11, NR = 34), the AUCs of this signature were 0.88 (0.85-0.91) and 0.99 (0.98-1), respectively (**Figures S12C, S12D**). For the CM009 cohort, we used its larger pretreatment dataset (38) (CM009_PRE, n.patients = 56, R = 9, NR = 47), and this signature possessed an AUC of 0.89 (0.85-0.93) (**Figure S12E**). In addition, two more independent ccRCC bulk RNA-seq datasets were utilized: PMC6035749 (46) and GSE67501 (47). For PMC6035749 (n.patients = 17, R = 5, NR = 12), the performance of ccRCC.Sig was AUC = 0.87 (0.79-0.96), sensitivity = 100% (78-100%), and specificity = 60% (19-92%) (**Figure 5C**). For GSE67501 (n.patients = 11, R = 4, NR = 7), ccRCC.Sig accurately distinguished all responders from nonresponders, with AUC = 1 (1–1), sensitivity = 100% (65-100%), and specificity = 100% (47-100%) (**Figure 5D**).

To further justify the predictive capability of ccRCC.Sig, we compared ccRCC.Sig with 13 other ICB response signatures previously reported (8, 48–58), including the recognized IMPRES signature (51). The details of these signatures are listed in **Supplementary Table 3**. They were used as independent classifiers to predict the ICB outcomes of six bulk RNA-seq cohorts using Cancerclass. The results show that ccRCC.Sig dramatically improved the performance of ICB response prediction in most cohorts (**Figure 5E**, **Figures S12F-S12I**, **Supplementary Table 4**). As a comparison, the performance of IMPRES.Sig ranked fourth in both the CheckMate and CM009_PRE cohorts (**Figure 5E**, **Figure S12I**) and fifth and third in the CM025 and CM010 cohorts (**Figures S12G, S12H**). We also compared ccRCC.Sig with clinical traits (e.g., age) and other molecular features (e.g., TMB, PD-1, tumor purity and angiogenesis, etc.), and the results showed the AUC of ccRCC.Sig was 0.91 (115 samples remained after deleting missing data samples) and that of other features was 0.48–0.62 (**Figure 5F**). In addition, we validated ccRCC.Sig using three more machine learning algorithms, i.e., support vector machine (SVM), naïve Bayes (NB) and k-nearest neighbors (KNN). As shown in **Figure 5G**, ccRCC.Sig still performed well in most cohorts. These results indicate that its predictive performance is stable.

In particular, prediction using Cancerclass is able to generate continuous prediction scores (z score) based on gene expression levels and convert them to probabilities (24). Therefore, to assess a patient’s resistance risk after ICB treatment, we converted the prediction scores to nonresponse probabilities and estimated the risk by logistic regression (**Figure S12J**). Based on the pretreatment RNA-seq data of tumor patients, we can estimate the resistance probability and provide guidance and a reference for patients to decide whether to accept ICB treatment by using this model.

Moreover, we explored the relationship between ccRCC.Sig and the prognosis of patients. Univariate Cox regression demonstrated the pretreatment GSVA score of ccRCC.Sig, PBRM1 mutation and intratumor heterogeneity (ITH) were closely related to poorer OS (**Figure 5H**), and multivariate Cox regression found that ccRCC.Sig, wild-type PBRM1 (20, 46, 59) and ITH were independent risk factors (**Figure S12K**).

We also found a relationship between ccRCC.Sig and a few known ICB response factors. The results showed that ccRCC.Sig was positively correlated with immune checkpoint molecules (PD-1, CTLA-4 and IDO1, **Figures S13A–S13C**) but not with TMB or ITH (**Figures S13D, S13E**). This is consistent with previous studies. Although TMB and ITH are associated with the ICB response in various tumors (11, 60–62), they are not associated with the ICB response in ccRCC (4, 7, 12, 13, 20). Angiogenesis (63, 64) and myeloid infiltration (7, 63) have been reported to be related to the ICB response and resistance in ccRCC, respectively. Our results show that ccRCC.Sig was negatively correlated with pro-response angiogenesis signaling (**Figure S13F**) but positively correlated with pro-resistant myeloid infiltration (**Figure S13G**).

## Discussion

Herein, we reanalyzed two publicly available ccRCC scRNA-seq datasets (12, 14) to explore effective predictive immunocyte subtypes and signatures in ccRCC. We identified three cell subtypes that were closely related to ICB outcomes: MKI67^+^ CD4Ts and MKI67^+^ Tregs contribute to ICB resistance, while antigen-presenting monocytes are correlated with the ICB response. We harnessed the gene markers associated with the above three subtypes and developed a signature, ccRCC.Sig. The prediction capability of ccRCC.Sig was systematically evaluated using various datasets, modeling methods and known risk factors. Our analyses demonstrated that compared to conventional prediction factors, ccRCC.Sig dramatically improved the reliability of ICB outcome prediction for ccRCC patients.

There are few ICB response prediction biomarkers currently available for ccRCC, and the predictive performance of these biomarkers varies among different tumor types (65). Immunohistochemistry (IHC) to detect PD-L1 expression on tumor cells or tumor-infiltrating immunocytes is the first clinically validated and the most widely used biomarker currently in ICB therapy (66). However, a lack of PD-L1 expression is considered an insufficient negative predictor of immunotherapy response, and patients with IHC PD-L1 (–) may also benefit from ICB in some clinical trials (67). Another biomarker is TMB, which is a measure of total tumor mutation. However, the clinical application of TMB detection is limited by high technical requirements, complex data and the need for bioinformatics experts. Next-generation sequencing (NGS) is immature and expensive. In addition, flow cytometry can be used for the development and detection of biomarkers. However, this method also has limitations of high cost, complex operations, high technical requirements and limited antibody availability in clinical application. The predictive biomarker we developed, ccRCC.Sig has high sensitivity and specificity and can be detected by real-time quantitative PCR, which enables it to be a highly reliable and clinically practical prediction tool.

Studies have pointed out that CD4^+^ T cells are necessary for effective antitumor immunity (68). CD4Ts exert antitumor activity through various direct and indirect mechanisms, including cytolysis of tumors and modulation of the tumor microenvironment (TME) (69). It can increase the activity and quality of B cells and cytotoxic T lymphocyte (CTL) responses *via* cell–cell and cell surface receptor interactions (70). Tregs, although a major subset of CD4Ts, differ from traditional CD4Ts and normally mediate immunosuppression and tolerance in homeostasis and inflammation (71). The consensus holds that Tregs in the TME exert an inhibitory effect against tumor immunity (72). In this study, MKI67^+^ CD4Ts and MKI67^+^ Tregs were identified as belonging to proliferative subgroups. They are two cell subtypes with similar specific functional characteristics, which are also similar to the proliferative CD8Ts described by Borcherding et al. (27). Although the effects of proliferative MKI67^+^ CD4Ts/Tregs on ICB outcomes have been reported in other tumor types (73, 74), this correlation has not been documented in ccRCC before.

In recent years, researchers have identified similar proliferative populations in many tumor types, such as melanoma (28, 48), lung cancer (75, 76), gastric cancer (73), head and neck cancer (74), and colon cancer (77), by scRNA-seq analysis or other experimental methods. The characteristics of these populations appear to be shared across different tumors (28), including ccRCC (27, 78). However, studies targeting these proliferative populations have mostly focused on CD8Ts. Interestingly, a proportion of hyperproliferative cells were also present in Tregs and CD4Ts, similar to exhausted/dysfunctional CD8Ts (28, 75). These CD8Ts have several common features, including active proliferation (MKI67^+^), high immune checkpoint or coinhibitory receptor expression (PD-1^+^, LAG3^+^, CTLA4^+^), terminal differentiation (EOMES^+^) and defective IFNγ production (28, 75, 76, 78). In esophageal squamous cell carcinoma, the proliferative CD4-C5-STMN1 cell subtype has the most exhausted features (79). Studies have shown that PD-1+ CD8Ts proliferate explosively after PD-1 blockade and impair the ICB response in chronic viral infections and cancers (76, 80). Similarly, a study of sarcoidosis showed that PD-1 blockade rescued the proliferation of CD4Ts (81). We also examined the expression of the above proliferative CD8Ts-associated characteristic genes in our MKI67^+^ CD4Ts and MKI67^+^ Tregs. We found that in these two cell subtypes, some immune checkpoint molecules, effector molecules and exhausted marker genes were preferentially enriched (**Figures S14A, S14C**). This finding indicates that they also appear to share these dysfunctional features with proliferative PD-1^+^ CD8Ts.

We also found that MKI67^+^ CD4Ts and MKI67^+^ Tregs belong to the PD-1^+^ cell population (**Figures S14B, S14D**). Consistently, a portion of Tregs have recently been found to express moderate levels of PD-1 (73, 74), in addition to activated and exhausted CD8Ts. PD-1^+^ Tregs show high proliferation and DNA metabolism features in head and neck cancer (74), and the proliferative activity of these cells with hyperprogressive disease (HPD) is significantly increased after ICB treatment in gastric cancer and compromises ICB treatment (73). PD-1 expressed in these cells might be a negative regulator of proliferation and immunosuppressive activity, while PD-1 blockade enhanced those of these cells.

Monocytes bridge the innate and adaptive immune response, with a paradoxical effect on tumors, involving multiple mechanisms, such as immune tolerance, angiogenesis, tumor cell metastasis, antitumor effector production, and antigen-presenting cell activation (82). These effects depend on monocyte plasticity under TME stimulation. It has been reported that circulating monocytes can infiltrate mucosa or inflammatory tissues and differentiate into monocyte-derived macrophages (mo-Macs) or monocyte-derived dendritic cells (mo-DCs) (83). Studies have shown that mo-Macs are powerful producers of proinflammatory cytokines (e.g., TNFα, interleukin, and IFNγ) (84), which are essential for immunocyte recruitment and immune response triggering. In addition, mo-Macs isolated from ascites are capable of cross-presenting antigens *via* specific pathways (85). In addition to MHC class II molecular genes, the expression levels of proinflammatory cytokines such as TNF, IFNγ, and IL18, as well as complement system genes such as C1QA, C1QB, and C3, were also significantly higher in our antigen-presenting monocytes (Mono_C0) than in the other monocyte subclusters (**Figure S14E**). Antigen processing and presentation, IFNγ signaling, and inflammatory response/regulation-related pathways were also dramatically enriched in this cell subtype (**Figure 4D**, Extended data 3). Based on the above characteristics, we speculated that Mono_C0 probably belongs to a monocyte subgroup similar to functional mo-Macs. This hypothesis is also supported by Lam et al. (86), who found that microbial signals program monocytes to inflammatory macrophages, characterized by high expression of MHC class II molecules and intermediate levels of F4/80 and CD68. This microbial-induced transformation can effectively improve the ICB response rate of melanoma.

In summary, our study not only provides an effective prediction model for ccRCC immunotherapy but also provides a pipeline for the development of therapy prediction models. Our method is applicable for prediction model development for various tumor therapies. There are still limitations in this study. We only described the relationship between the above three cell types and ICB outcomes but did not clarify their mechanism. In the future, large sample experiments with more rigorous designs are needed to explore the mechanisms of these cells to consolidate the biological findings in this study.

## Methods

### scRNA-seq and bulk RNA-seq datasets collection

To explore immunocyte subtypes and signatures with good predictive capability for ICB outcomes, we downloaded and reanalyzed two available ccRCC scRNA-seq datasets published in the initial article (12, 14) and validated the results in multiple ccRCC pretreatment bulk RNA-seq datasets. The first scRNA-seq dataset (Bi’s dataset) (14) was obtained from fresh biopsy or surgical resection samples of 8 patients, of which 5 patients were treated with ICB (anti-PD-1 combined with or without TKIs/anti-CTLA-4), and the other 3 patients did not receive systemic treatment. According to RECIST v1.1 (21), two of the five ICB-treated patients had PR efficacy, and the other three had SD, PD, and NE efficacy. In this study, the NE sample (P912) was omitted throughout. Bi’s dataset is available from the Single Cell Portal (https://singlecell.broadinstitute.org/single_cell/study/SCP1288/tumor-and-immune-reprogramming-during-immunotherapy-in-advanced-renal-cell-carcinoma#study-summary). The second scRNA-seq dataset (Au’s dataset) (12) was obtained from nephrectomy samples from 2 patients treated with nivolumab with RECIST efficacy of PD and PR. The single-cell count matrices and metadata can be downloaded publicly from the University College London (UCL) website (https://doi.org/10.5522/04/16573640.v1). Detailed clinicopathological information for all patients in Bi’s dataset and Au’s dataset, as well as the processing, clustering and cell type definition methods of the scRNA-seq datasets, were described in detail in their original articles (12, 14).

The ICB pretreatment bulk RNA-seq data used for validation consisted primarily of CheckMate 009 (CM009, NCT01358721) (45), CheckMate 010 (CM010, NCT01354431) (44), CheckMate 025 (CM025, NCT01668784) (5) and Javelin101 (NCT02684006) (4) cohorts. The first three are prospective phase I, phase II, and phase III clinical trials of nivolumab in the treatment of advanced ccRCC. The CheckMate cohort was an integrated dataset of the above three bulk RNA-seq datasets selected against certain criteria, leaving 181 nivolumab-treated samples after the removal of the everolimus-treated samples. This cohort included 16 CM009 samples (R = 3, NR = 13), 45 CM010 samples (R = 11, NR = 34), and 120 CM025 samples (R = 25, NR = 86, NE = 9). The selection strategy and the integration, alignment, quantification, and batch effect correction methods for this dataset have been described in detail by Braun et al. (20), and the normalized RNA-Seq expression data are provided in their supplementary information. The Javelin101 cohort is a randomized phase III clinical trial (NCT02684006) of avelumab (anti-PD-L1) plus axitinib (TKI) versus sunitinib (multitarget TKI) for the treatment of ccRCC (4). This cohort includes pretreatment bulk RNA-seq data from a total of 726 patients, 354 of whom received avelumab plus axitinib and 372 of whom received sunitinib, and its normalized RNA-seq data can be found in the supplementary original article. In addition, we downloaded the CM009 cohort dataset, which has a larger number of patients (38) and can be subdivided into three datasets: a pretreatment dataset (CM009_PRE, n.patients = 59, R = 9, NR = 47, NE = 3), a week 4 treatment dataset (CM009_POST, n.patients = 55, R = 5, NR = 47, NE = 3), and a pretreatment and week 4 treatment paired samples dataset (CM009_Paired, n.patients = 42, R = 5, NR = 37). They are publicly available from ArrayExpress (accession number: E-MTAB-3218). Two other independent datasets of anti-PD-1 treatment were also downloaded to test the predictive performance of ccRCC.Sig, GSE67501 cohort (47), available on Gene Expression Omnibus (GEO), and PMC6035749 cohort (46), available from supplementary of original article. The database used in our study included MSigDB v7.5.1 (23) (http://www.gsea-msigdb.org/gsea/index.jsp), from which the Hallmark, KEGG, GOBP, and Reactome gene sets were downloaded for GSEA.

Detailed metadata for all scRNA-seq and bulk RNA-seq dataset samples used in this study can be found in **Supplementary Table 1**.

### Study design

According to the flow shown in **Figure S1A**, we extracted a total of 13 cell types from two scRNA-seq datasets. We then used scCODE v1.0.0.0 R package provided by Zou et al. (22) to identify the DE genes of responders and nonresponders in the above 13 cell types, respectively. scCODE can check the selected DE genes through a variety of testing methods, improving the accuracy of single-cell DE analysis. In this way, two DE gene lists, which were remarkably highly expressed in R and NR, were obtained for each cell type, with a total of 26 DE gene lists (Extended data 1. We then used the Investigate Gene Sets tool (http://www.gsea-msigdb.org/gsea/msigdb/annotate.jsp) to compute enriched gene sets between our gene lists and gene sets in MSigDB (23). Due to this tool’s limitation on the submitted gene numbers (≤ 500), for the 26 DE gene lists identified by scCODE, we sorted them according to the absolute value of logFC of each DE gene list from large to small. The gene sets enriched in GOBP, Hallmark, KEGG and Reactome were identified by submitting the top 500 genes (all genes were submitted if less than 500). With the default settings of this tool (show top 10 genes & FDR q-value less than 0.05), each DE gene list can obtain several gene sets enriched in GOBP, Hallmark, KEGG and Reactome. The predictive capability of these gene sets was tested using Cancerclass v1.34.0 R package (24). Due to the limitation of this package on the input gene numbers (minimum 3 genes), we excluded gene sets with fewer than 3 genes. Eventually, 1008 gene sets were obtained for subsequent analysis (Extended data 2).

We tested the predictive capability of the above 1008 gene sets on the ICB outcomes of the CheckMate cohort (n.patients = 72, R = 39, NR = 133) using the Cancerclass R package. The prediction sensitivity and specificity were assessed by ROC curve and corresponding AUC. This R package is dedicated to developing and validating classification tests for high-dimensional molecular data. Feature selection and nearest centroid classification were performed in sequence. Finally, the classification results were carefully verified by using the multiple random validation protocol to generate continuous prediction scores (24). Each gene set was tested as an independent classifier, and the p value of the ROC curve was calculated by Welch’s t test built into the Cancerclass R package, which reflects the effectiveness of the classifier’s classification test results. These 1008 ROC p values (Extended data 2) were used for subsequent sorting of cell subtypes.

### Single-cell RNA sequencing data processing

We directly extracted the cell types defined by the authors in the original articles of Bi’s dataset (14) and Au’s dataset (12). For subtype analysis of each cell type, we performed fine clustering utilizing Seurat v4.0.4 R package (25). Specifically, we first performed across-sample integration on the extracted datasets. Prior to this, the expression of each gene was normalized by the total expression in the corresponding cells, multiplied by a scaling factor of 10,000, and then log2 transformed (Seurat default method). Two thousand variable features were identified by FindVariableFeatures and used for subsequent analysis. Then, the mutual nearest neighbors (MNN) method of the batchelor v1.0.1R package (87) was used to correct batch effects, and ScaleData were used to regress the percentage of mitochondrial transcripts. The integrated assay was used only for dimension reduction and clustering, and the raw log-normalized expression data were used for all DE and gene level analyses. Then, principal component analysis (PCA) was performed on the integrated assay by using RunPCA. The first 20 principal components were taken for Louvain clustering of cells with a resolution parameter of 0.5. Finally, visualization was performed in two-dimensional space by uniform manifold approximation and projection (UMAP) (Dims = 1:20). This procedure was used for scRNA-seq datasets from all cell type analyses.

### Gene differential expression analysis

Using the FindAllMarkers built in the Seurat package, DE analysis was performed on each Louvain cluster and all other clusters by setting the parameters min.pct = 0.1 and logfc.threshold = 0.25. Genes with p.adjust < 0.05 were selected as cluster-specific marker genes. Using the FindMarkers built into the Seurat package, we compared all gene expression fold changes in nonresponders and responders in corresponding cells by setting the parameter logfc.threshold = -Inf, min.pct = -Inf, min.diff.pct = -Inf. Utilize the default settings of scCODE v1.0.0.0 The R package (22) (https://github.com/XZouProjects/scCODE) was used to analyze the DE genes of responders and nonresponders in each cell type. The generalized linear model method of Limma v3.46.0 The R package (88) (https://doi.org/doi:10.18129/B9.bioc.limma) was used to compute the gene expression fold changes of nonresponders and responders in the CheckMate cohort. We fit the linear model of each gene using the weighted least square method through the lmFit function and compared each gene through the contrasts.fit function. Finally, we used the empirical Bayes smoothing of standard errors. In all DE analyses, genes with Bonferroni FDR-corrected p values < 0.05 were considered DE genes by the bilateral Wilcoxon rank sum test.

### GSVA, GSEA and IPA

We applied the GSVA method with default settings to assign a specific gene signature activity score for individual cells or samples, as implemented in GSVA v1.38.2 R Package (26) (https://doi.org/doi:10.18129/B9.bioc.GSVA). GSEA was performed on preordered DE gene lists based on predownloaded Hallmark, KEGG, GOBP, and Reactome gene sets using the default parameters of clusterProfiler v3.18.1 R package (89) (https://doi.org/doi:10.18129/B9.bioc.clusterProfiler). This R package can also be used to examine whether a particular gene set is enriched at the top or bottom of a preordered gene list. Gene sets with FDR-corrected p values < 0.05 by the Benjamini–Hochberg method were considered to be significantly enriched in one group when two groups were compared. Ingenuity pathway analysis was performed using QIAGEN IPA software (IPA Spring Release April 2022, [https://digitalinsights.qiagen.com/products-overview/discovery-insights-portfolio/analysis-and-visualization/qiagen-ipa/]) with lists of specific marker genes in the corresponding clusters as detected by Seurat.

### Survival analysis, Cox and logistic regression analysis

Kaplan–Meier method-based survival analysis and univariate and multivariate Cox regression were performed on GSVA scores for specific gene signatures using Survival v3.2-13 R package and SurvivMiner v0.4.9 R package. To define “high-score” and “low-score” groups, in the CheckMate cohort, the groups were dichotomized at the median GSVA score. For other bulk RNA-seq datasets, we calculated the optimal cutpoint using the surv_cutpoint function built in the Survminer R package. The groups were compared using the log-rank test. Logistic regression analysis was performed using the R software embedded glm function for ICB outcomes and mean expression of specific gene signatures. The corresponding ROC curves were drawn by the pROC v1.18.0 R package.

### Workflow of ICB response prediction signature development

Based on the cluster-specific genes of MKI67^+^ CD4T, MKI67^+^ Treg and Mono_C0 subtypes, we developed our gene signatures using Cancerclass v1.34.0 R package according to the flow shown in **Figure S8**. Specifically, the p values of ROC curves (30 in total) using the enriched GOBP gene sets of these three cells were obtained using Cancerclass v1.34.0 R package and then FDR-adjusted with the Benjamini–Hochberg method (Extended data 5). We united all the gene sets with p.adjust < 0.05 (26 gene sets in total) to obtain a total of 493 genes (union.genelist). Meanwhile, we compared all gene expression fold changes in NR and R through DE analysis in the corresponding cell subtypes. For MKI67^+^ CD4Ts, we selected the NR vs. R genes avg_log2FC > -0.1; for MKI67^+^ Tregs, we selected the NR vs. R genes avg_log2FC > -0.3; and for Mono_C0, we selected the NR vs. R genes avg_log2FC < 0.1. After intersecting these selected genes, we obtained a gene list of 11,884 genes (intersect.genelist). Subsequently, we took the intersection of union.genelist and intersect.genelist and excluded absent genes in the CheckMate cohort. We finally obtained the “genelist”, which contained 209 genes. Next, the genelist was used to execute the cycle algorithm shown in **Figure S8** (purple box). First, we randomly selected combinations containing 208 genes from the genelist, resulting in 209 different combinations. The predictive capability of all these combinations was then tested, and the AUC was estimated in the CheckMate cohort using the Cancerclass R package. The gene combination with the largest AUC (genelist_maxAUC_) of these 209 combinations was preserved and used for the next cycle. The cycle was repeated until the total gene number of the gene combination was < 3, and finally, all the circulating genelist_maxAUC_s and their corresponding AUCs were output (Extended data 6). A combination with appropriate gene numbers was selected as ccRCC.Sig for subsequent analysis.

### Statistical analysis

The predictive capability of each classifier in this study for ICB outcomes was assessed by plotting the ROC, calculating the AUC, and estimating the sensitivity and specificity implemented in Cancerclass v1.34.0 R package24, and its effectiveness was assessed by the ROC curve’s p value. The 95% confidence intervals for sensitivity and specificity were calculated by Wilson’s method built into Cancerclass v1.34.0 R package, and p-values were calculated by Welch’s t-test. Unless otherwise noted, all p-values in this manuscript were adjusted by the Benjamini–Hochberg method, and adjusted p-values < 0.05 were considered statistically significant. Wilcoxon’s test was used to classify variables. All confidence intervals were reported as binomial 95% confidence intervals. All statistical analyses in this study were performed with R v3.5.3 software.

## Data availability statement

The original contributions presented in the study are included in the article/**Supplementary Material**. Further inquiries can be directed to the corresponding authors. The computer R codes for processing and analysis of this study are available at https://github.com/XZouProjects/ccRCC.Sig.

## Author contributions

Conceptualization: XH and XZ. Methodology: XZ and JH. Investigation: XZ and KZ. Project administration: XH and XZ. Writing – original draft: KZ, LG, JH, XZ and XH. All authors contributed to the article and approved the submitted version.

## Funding

This work was supported in part by the National Natural Science Foundation of China [82170045 to JH]; the Innovative Research Team of High-level Local Universities in Shanghai [SHSMU-ZLCX20212301 to JH] and the Shanghai Municipal Health Commission Project [202040375 to XH].

## Conflict of interest

The authors declare that the research was conducted in the absence of any commercial or financial relationships that could be construed as a potential conflict of interest.

## Publisher’s note

All claims expressed in this article are solely those of the authors and do not necessarily represent those of their affiliated organizations, or those of the publisher, the editors and the reviewers. Any product that may be evaluated in this article, or claim that may be made by its manufacturer, is not guaranteed or endorsed by the publisher.
