# Supplementary material for: An immunotherapy response prediction model derived from proliferative CD4+ T cells and antigen-presenting monocytes in ccRCC
Source: Front Immunol. 2022 Aug 25;13:972227. doi: 10.3389/fimmu.2022.972227 (PMC9452905; doi:10.3389/fimmu.2022.972227)
Supplement: Supplementary file 1 [file DataSheet_1.pdf]

Supplementary Material

Supplementary Figures and Tables

1.1 Supplementary Figures

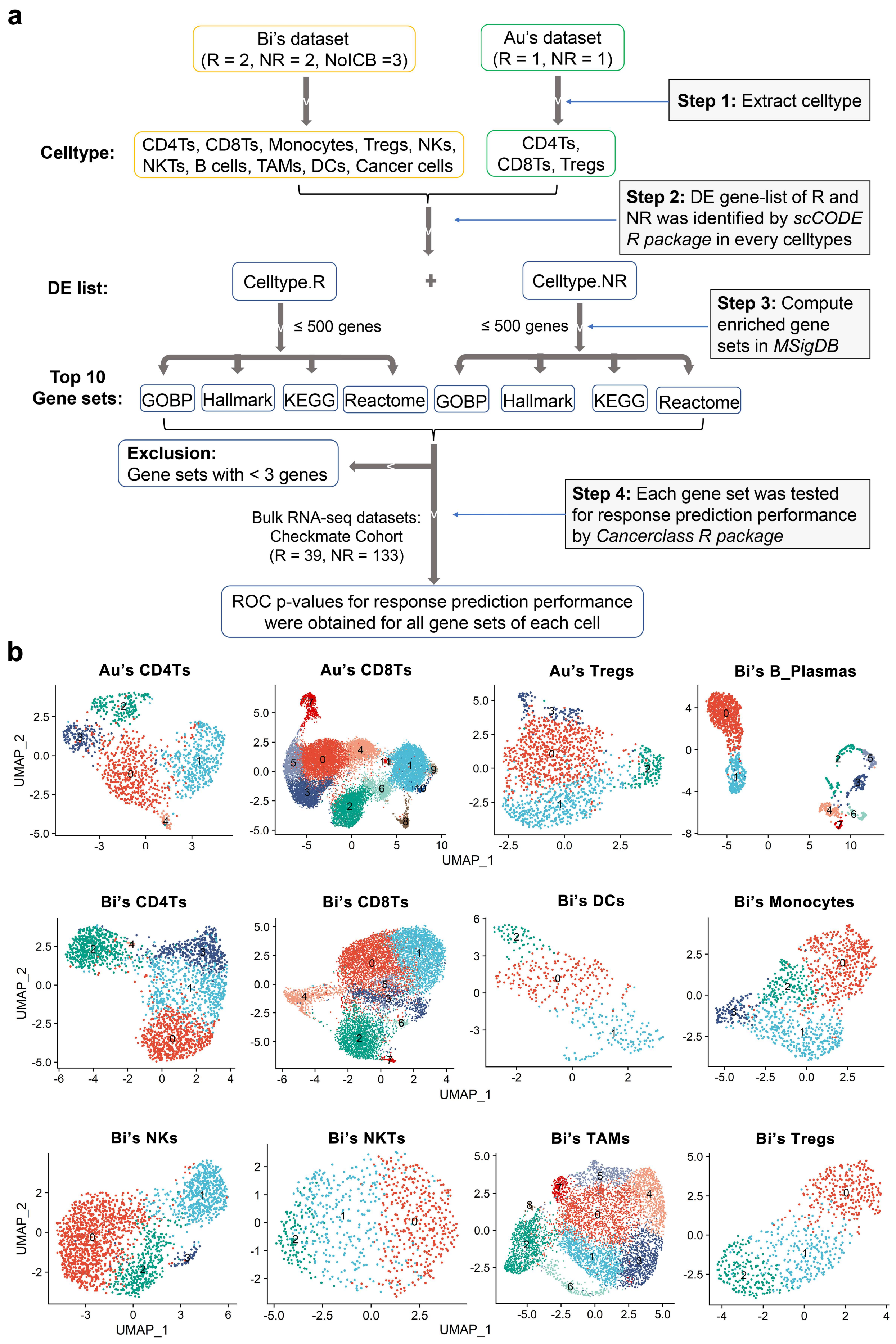

Supplementary Figure 1. The workflow of ICB response prediction-related cell types identification and cell subtype analysis of Au's and Bi's scRNA-seq datasets. **(a)** The workflow for identifying ICB response prediction-related cell types. CD4Ts, $CD4^+$ T cells; CD8Ts, $CD8^+$ T cells; Tregs, regulatory T cells; DCs, dendritic cells; NKs, natural killer cells; NKTs, natural killer T cells; TAMs, tumor-associated macrophages. Immune checkpoint blockade therapy (ICB) outcomes: R, responders; NR, nonresponders. **(b)** Uniform manifold approximation and projection (UMAP) of 12 immune cell types extracted from Bi's and Au's datasets.

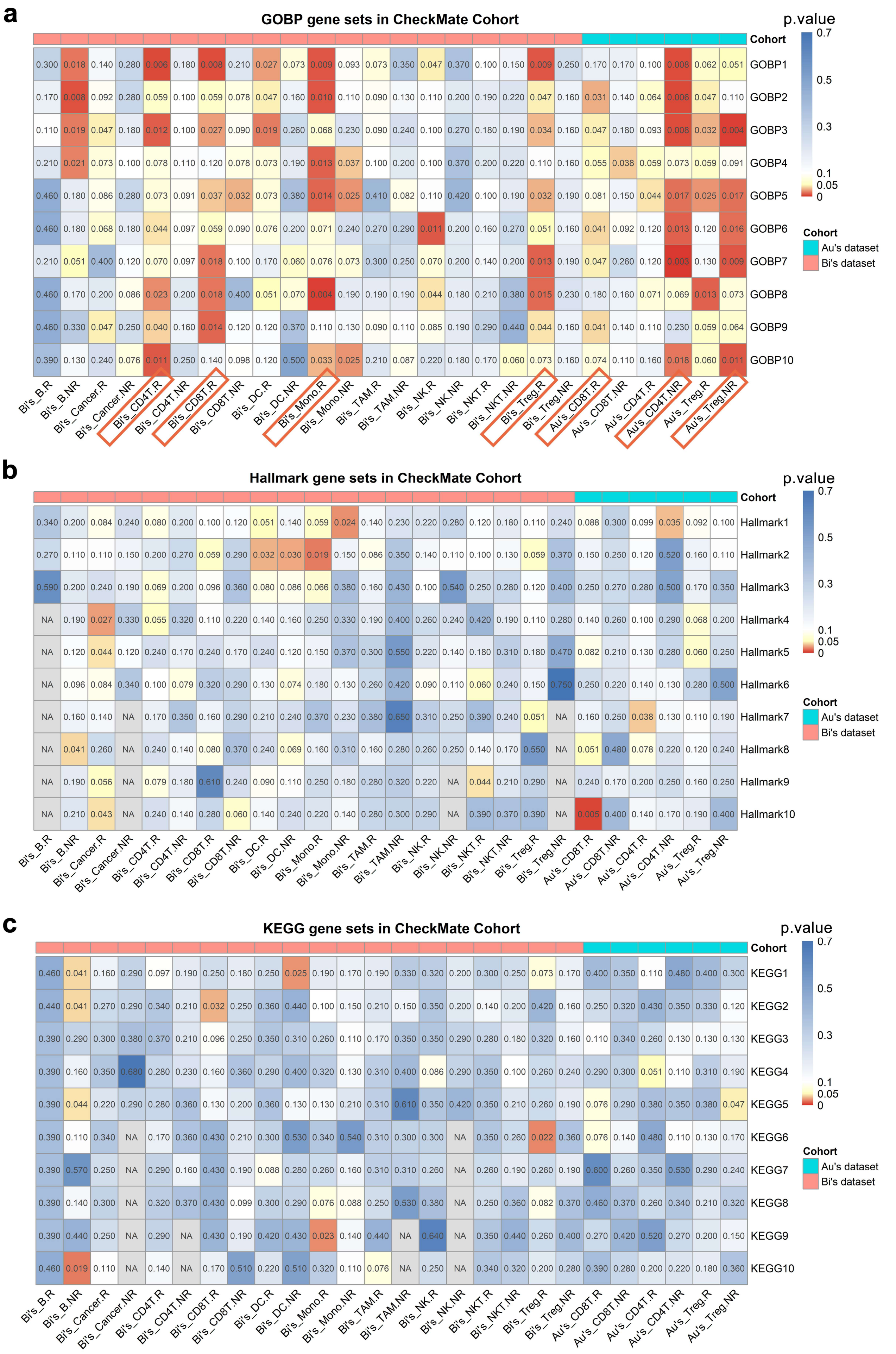
a
GOBP gene sets in CheckMate Cohort
p.value
0.7
0.5
0.3
0.1
0.05
0
Cohort
Au's dataset
Bi's dataset
GOBP1
GOBP2
GOBP3
GOBP4
GOBP5
GOBP6
GOBP7
GOBP8
GOBP9
GOBP10
Bi's_B.R
Bi's_B.NR
Bi's_Cancer.R
Bi's_Cancer.NR
Bi's_CD4T.R
Bi's_CD4T.NR
Bi's_CD8T.R
Bi's_CD8T.NR
Bi's_DC.R
Bi's_DC.NR
Bi's_Mono.R
Bi's_Mono.NR
Bi's_TAM.R
Bi's_TAM.NR
Bi's_NK.R
Bi's_NK.NR
Bi's_NKT.R
Bi's_NKT.NR
Bi's_Treg.R
Bi's_Treg.NR
Au's_CD8T.R
Au's_CD8T.NR
Au's_CD4T.R
Au's_CD4T.NR
Au's_Treg.R
Au's_Treg.NR
b
Hallmark gene sets in CheckMate Cohort
Hallmark1
Hallmark2
Hallmark3
Hallmark4
Hallmark5
Hallmark6
Hallmark7
Hallmark8
Hallmark9
Hallmark10
c
KEGG gene sets in CheckMate Cohort
KEGG1
KEGG2
KEGG3
KEGG4
KEGG5
KEGG6
KEGG7
KEGG8
KEGG9
KEGG10

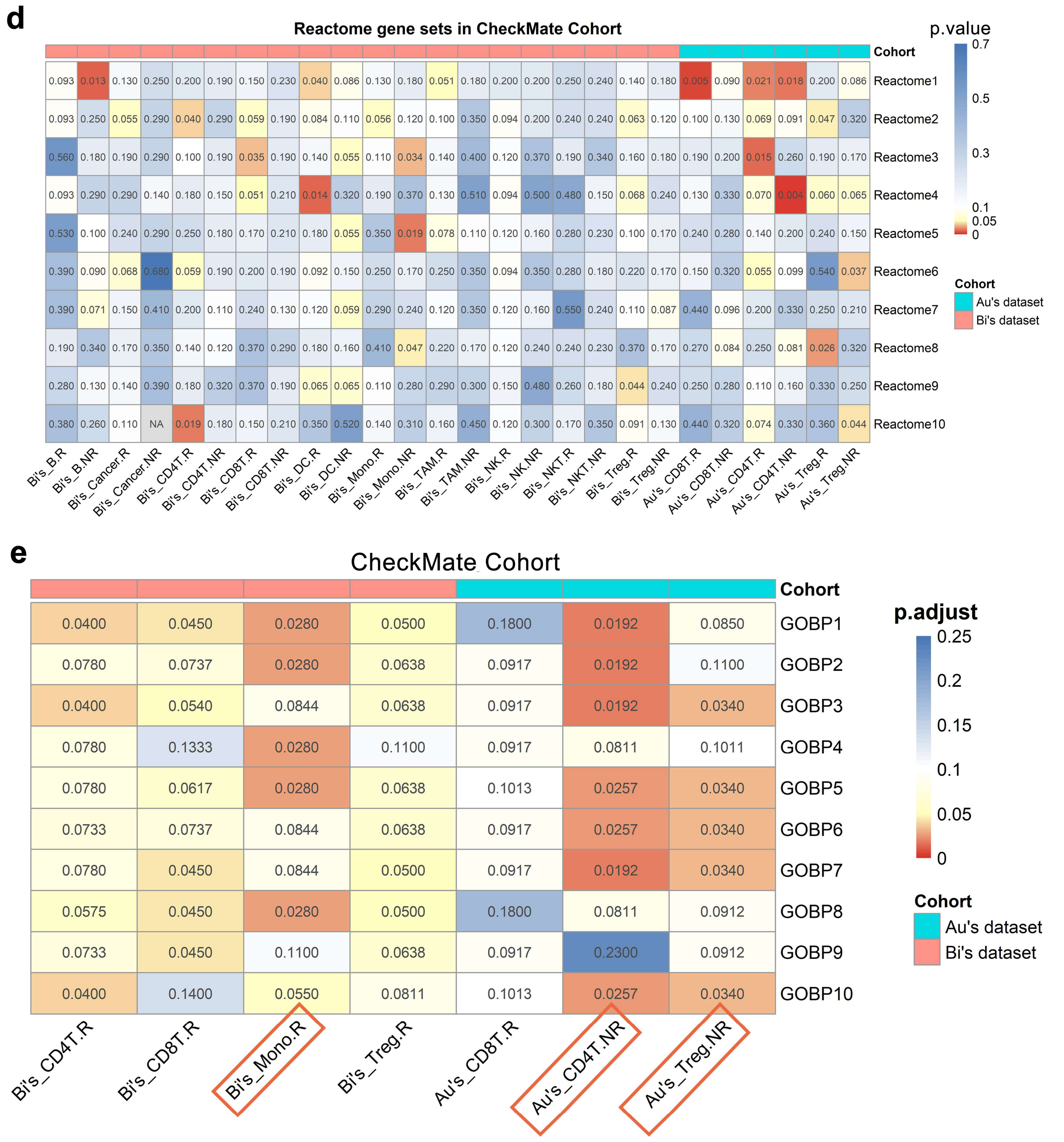

Supplementary Figure 2. The performances of 26 DE gene list-enriched gene sets for predicting ICB outcomes and identification of ICB response-related cell types. Au's and Bi's scRNA-seq datasets and CheckMate cohort (n.patients = 172, R = 39, NR = 133) were analyzed. All the pathways' details can be obtained from Extended data 3. **(a)-(d)** P values of receiver operating characteristic (ROC) curves for predicting ICB outcomes of 26 DE gene-list enriched **(a)** GOBP, **(b)** Hallmark, **(c)** KEGG and **(d)** Reactome gene sets. Immune checkpoint blockade therapy (ICB) outcomes: R, responders; NR, nonresponders. **(e)** Adjusted p values of ROC curves of gene sets enriched in seven significant DE gene lists. P values were FDR-adjusted by the Benjamini-Hochberg method.

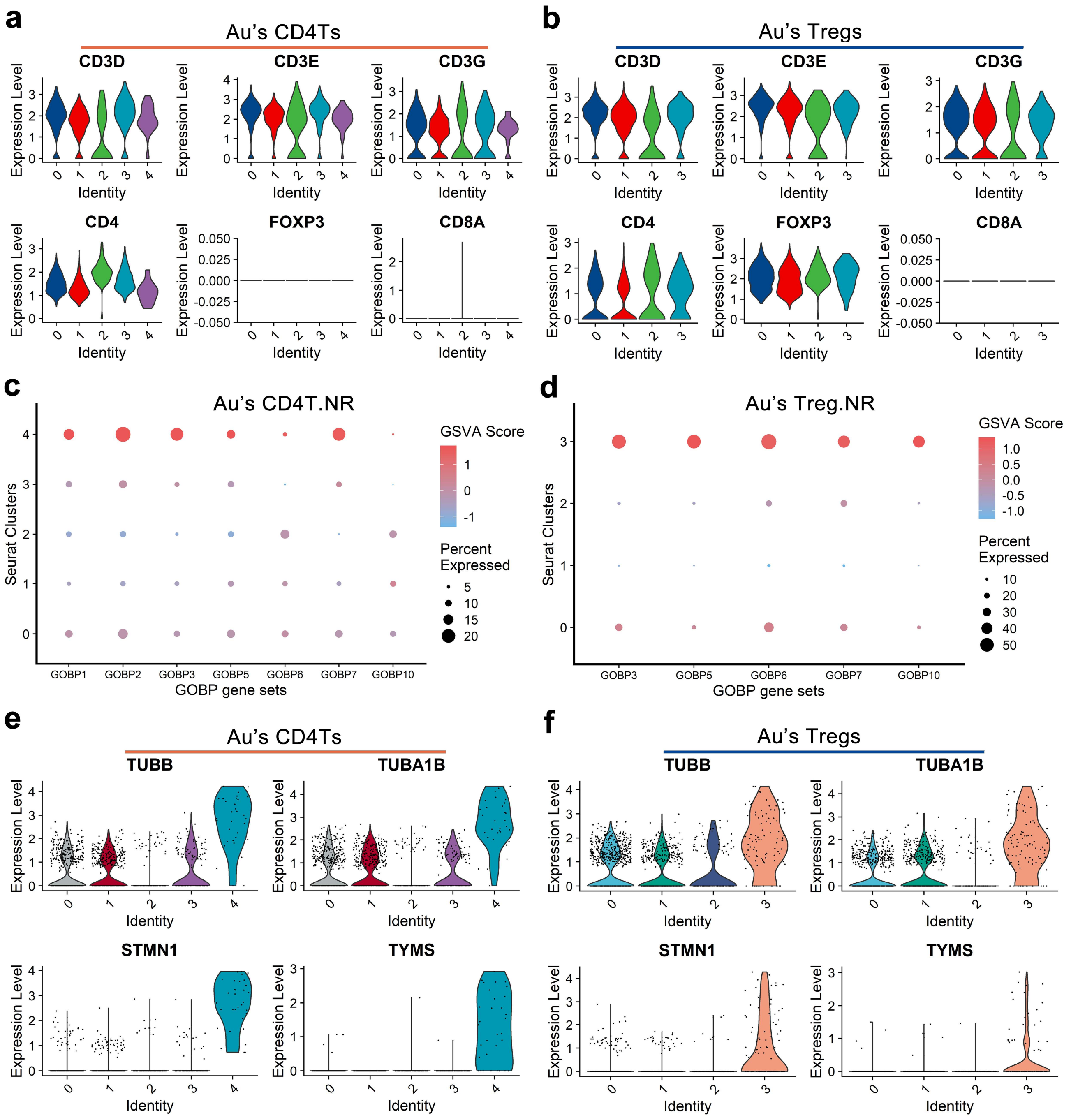
a
Au's CD4Ts
CD3D
CD3E
CD3G
CD4
FOXP3
CD8A
Expression Level
Identity
b
Au's Tregs
c
Au's CD4T.NR
Seurat Clusters
GSVA Score
Percent Expressed
GOBP1
GOBP2
GOBP3
GOBP5
GOBP6
GOBP7
GOBP10
GOBP gene sets
d
Au's Treg.NR
e
TUBB
TUBA1B
STMN1
TYMS
f

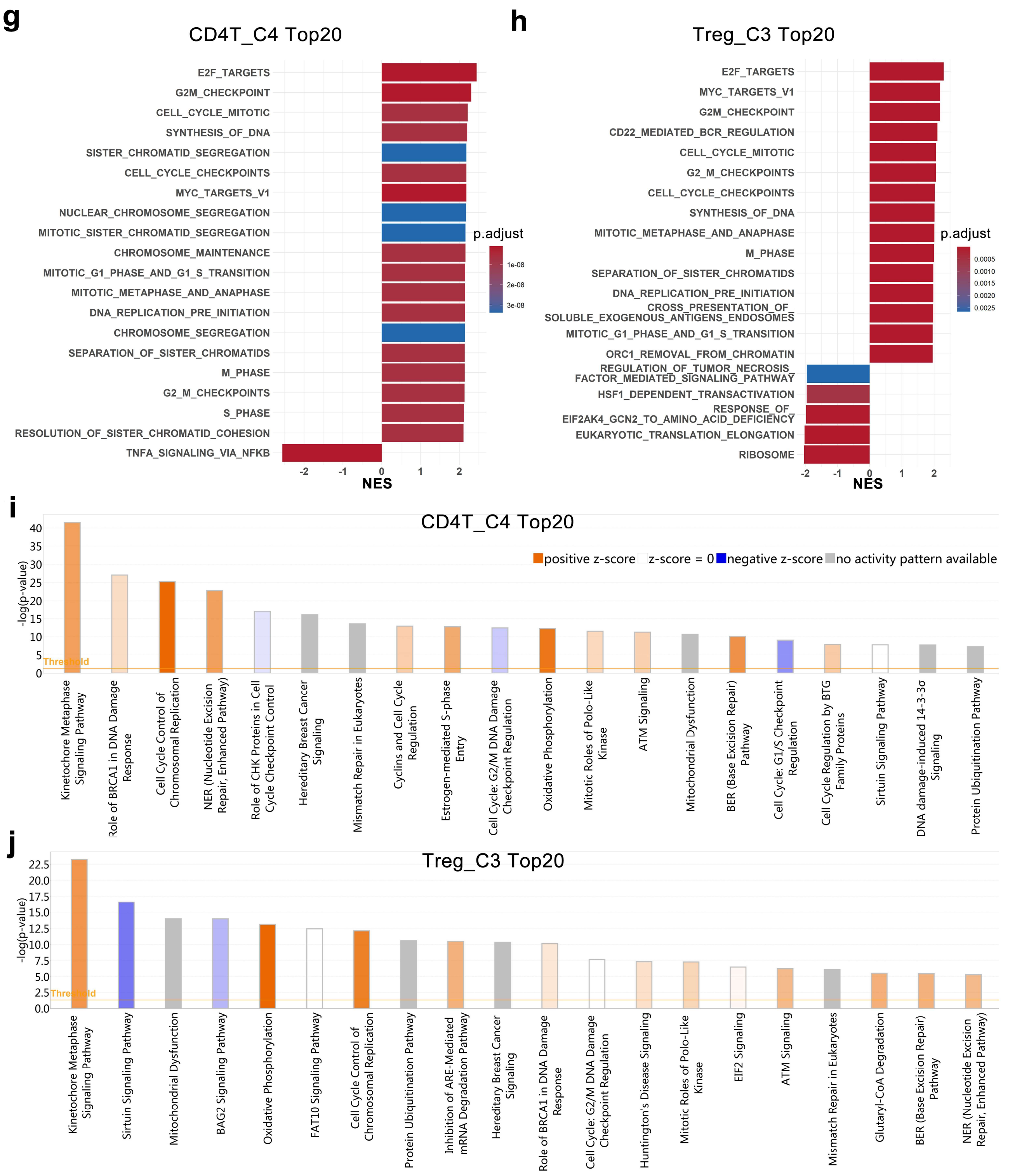

Supplementary Figure 3. Proliferative subtypes of CD4Ts and Tregs were enriched in nonresponders and associated with ICB resistance. The Au's CD4Ts and Treg scRNA-seq datasets were analyzed. Related to Fig. 2. **(a)** and **(b)** Violin plot of the expression levels of classical CD4T and Treg marker genes in Au's CD4Ts and Treg subclusters. CD3D, CD3E, CD3G and CD4 are both CD4Ts and Treg markers, FOXP3 is the specific marker gene of Tregs, and CD8A is the CD8Ts marker gene. **(c)** and **(d)** ICB response prediction-related CD4Ts and Treg subtypes were identified by locating the effective (ROC p.adjust < 0.05) predictive gene sets expression via the gene set variation analysis (GSVA). Related to Fig. S2e and Extended data 2. **(e)** and **(f)** Violin plot of the expression levels of proliferative marker genes in Au's CD4Ts and Treg subclusters. **(g)** and **(h)** GOBP, Hallmark, KEGG and Reactome analysis results of Au's CD4Ts subcluster 4 (CD4T_C4) and Au's Treg subcluster 3 (Treg_C3) compared with other subclusters. The top 20 pathways sorted by the absolute value of the normalized enrichment score (NES) are listed. **(i)** and **(j)** Ingenuity pathway analysis (IPA) results of CD4T_C4 and Treg_C3 compared with other subclusters. The top 20 pathways sorted by the p-value are listed. Orange bars, activated; blue bars, suppressed.

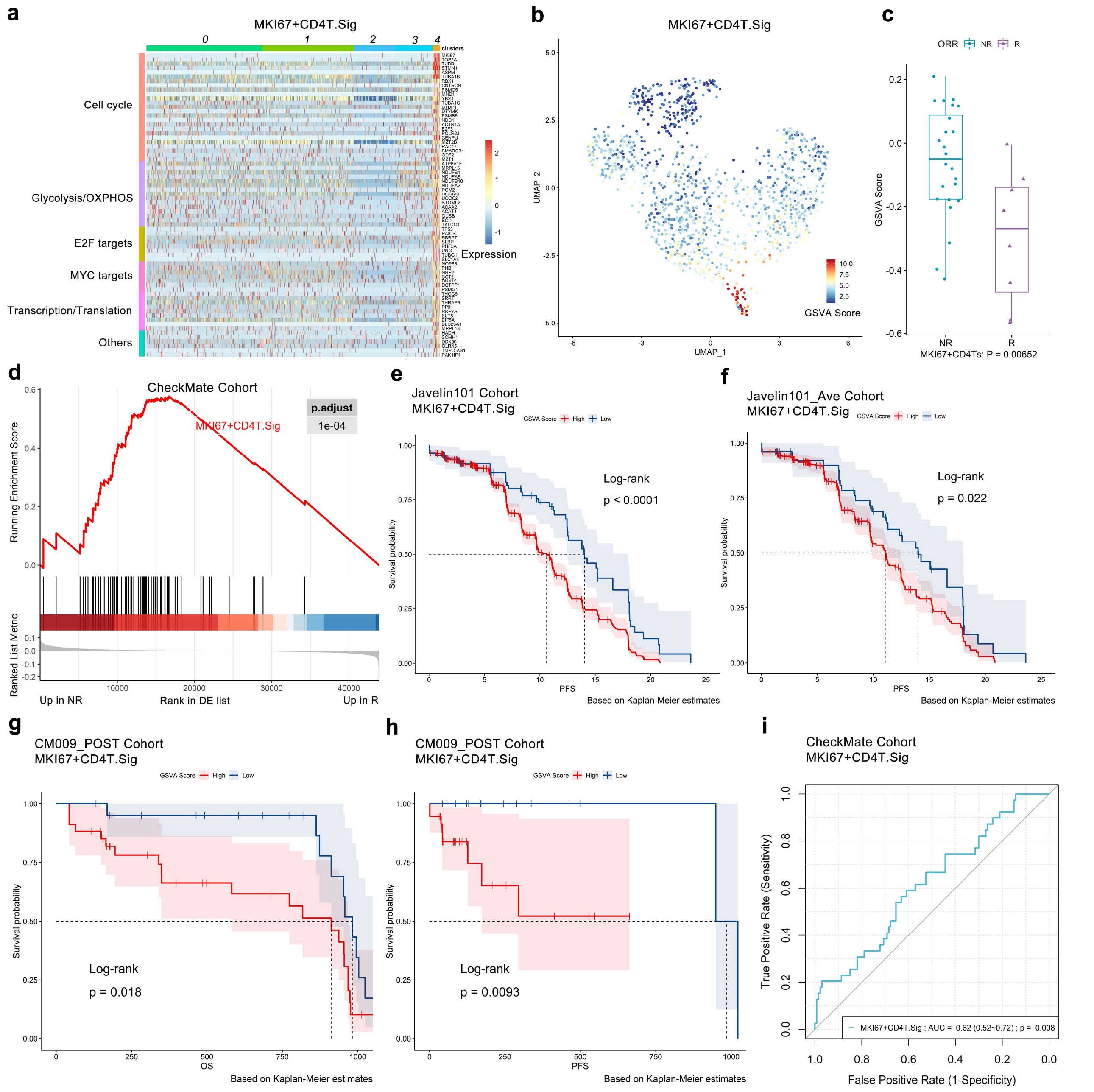

Supplementary Figure 4. A 70-gene signature penal that can specifically characterize the proliferative subtype of CD4Ts and correlated with ICB resistance. The scRNA-seq dataset - Au's CD4Ts and bulk RNA-seq datasets – CheckMate, Javelin101 and CM009_POST were analyzed. Related to Fig. 3. **(a)** Heatmap of the expression of a 70-gene signature (MKI67^{+}CD4T.Sig) representing the highly enriched pathways of the proliferative subtype of CD4Ts (MKI67^{+} CD4Ts). **(b)** Feature plot of MKI67^{+}CD4T.Sig GSVA scores showed that it can specifically characterize MKI67^{+} CD4Ts. **(c)** Boxplot discovered that MKI67^{+}CD4T.Sig GSVA scores of NR were significantly higher than that of R in MKI67^{+} CD4Ts via GSVA. Center line, median. Box limits, upper and lower quartiles. Whiskers, 1.5 interquartile range. Points beyond whiskers, outliers. A two-sided Wilcoxon test was used to determine significance. **(d)** GSEA validated that MKI67^{+}CD4T.Sig was enriched in NR of the CheckMate cohort (n.patients = 172, R = 39, NR = 133). The P value was FDR-adjusted by the Benjamini–Hochberg method. **(e)-(h)** Survival analysis was performed on GSVA scores for MKI67^{+}CD4T.Sig in pretreatment **(e)** Javelin101 cohort (n.patients = 726), **(f)** avelumab plus axitinib treated Javelin101 cohort (n.patients = 354), and **(g, h)** posttreatment CheckMate 009 (CM009_POST) cohort (n.patients = 55, R = 5, NR = 47, NE = 3) based on Kaplan–Meier method. The groups were dichotomized at the optimal cutoff point of the GSVA score, and the log-rank test was used to determine significance. Dashed line: median survival time. Color range: 95% confidence interval (CI). **(i)** The performance of univariate logistic regression model of MKI67^{+}CD4T.Sig for predicting ICB outcomes.

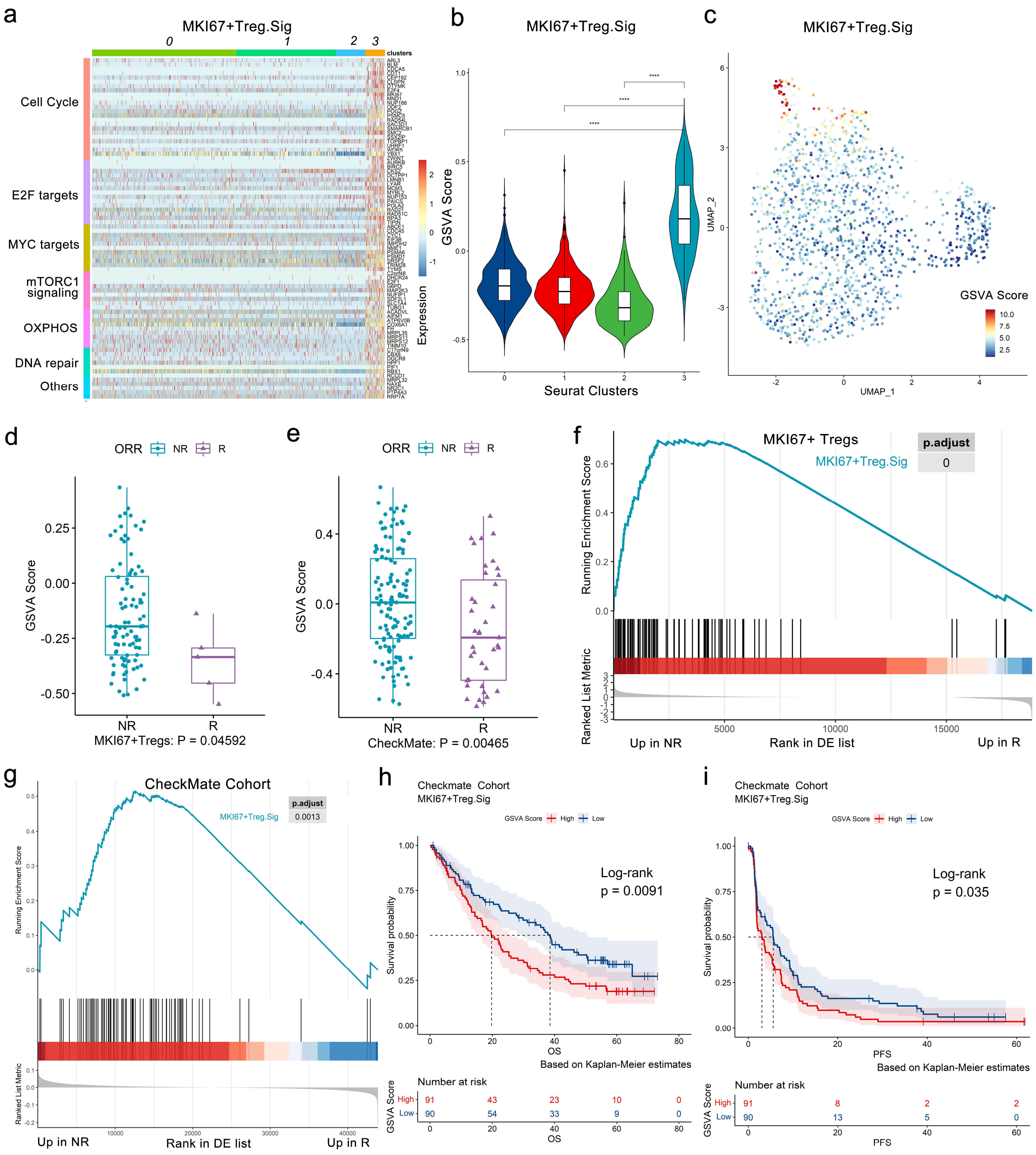

a
MKI67+Treg.Sig
Cell Cycle
E2F targets
MYC targets
mTORC1 signaling
OXPHOS
DNA repair
Others
Expression
b
MKI67+Treg.Sig
GSVA Score
Seurat Clusters
c
MKI67+Treg.Sig
GSVA Score
UMAP_1
UMAP_2
d
ORR NR R
GSVA Score
MKI67+Tregs: P = 0.04592
e
ORR NR R
GSVA Score
CheckMate: P = 0.00465
f
MKI67+ Tregs
p.adjust
MKI67+Treg.Sig 0
Running Enrichment Score
Ranked List Metric
Up in NR
Rank in DE list
Up in R
g
CheckMate Cohort
p.adjust
MKI67+Treg.Sig 0.0013
Up in NR
Rank in DE list
Up in R
h
Checkmate Cohort
MKI67+Treg.Sig
Log-rank
p = 0.0091
Survival probability
OS
Based on Kaplan-Meier estimates
Number at risk
i
Checkmate Cohort
MKI67+Treg.Sig
Log-rank
p = 0.035
PFS
Based on Kaplan-Meier estimates
Number at risk

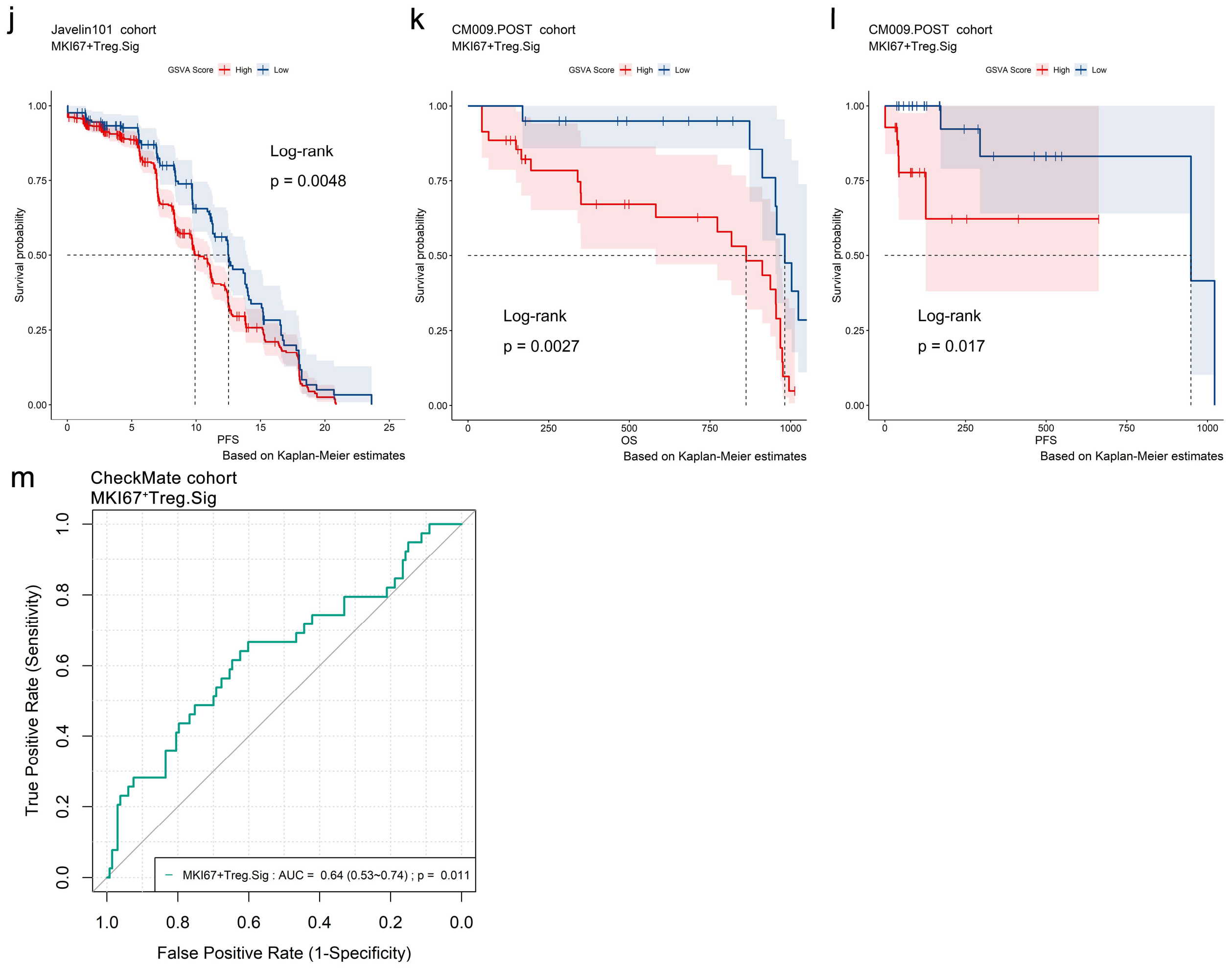

Supplementary Figure 5. Validation of the MKI67+ Treg signature using independent bulk RNA-seq datasets. The scRNA-seq dataset - Au's Tregs and bulk RNA-seq datasets – CheckMate, Javelin101 and CM009_POST – were analyzed. **(a)** Heatmap of the expression of an 80-gene signature penal (MKI67$^+$Treg.Sig) representing the highly enriched pathways of the proliferative Treg subtype (MKI67$^+$ Tregs). **(b)** and **(c)** Violin and feature plot of MKI67$^+$Treg.Sig GSVA scores showed that it can specifically characterize MKI67$^+$ Tregs. Center line, median. Box limits, upper and lower quartiles. Whiskers, 1.5 interquartile range. Points beyond whiskers, outliers. A two-sided Wilcoxon test was used to determine significance. ****P < 0.0001. **(d)** and **(e)** Boxplot discovered and validated that MKI67$^+$Treg.Sig GSVA scores of NR were significantly higher than those of R in both **(d)** MKI67$^+$ Tregs and **(e)** the CheckMate cohort (n.patients = 172, R = 39, NR = 133) via GSVA analysis. Center line, median. Box limits, upper and lower quartiles. Whiskers, 1.5 interquartile range. Points beyond whiskers, outliers. A two-sided Wilcoxon test was used to determine significance. **(f)** and **(g)** GSEA analysis discovered and validated that MKI67$^+$Treg.Sig was enriched in NR of **(f)** MKI67$^+$ Tregs and **(g)** the CheckMate cohort (n.patients = 172, R = 39, NR = 133). The p value was FDR-adjusted by the Benjamini–Hochberg method. **(h)-(l)** Survival analysis was performed on GSVA scores for MKI67$^+$Treg.Sig in the pretreatment **(h, i)** CheckMate cohort (n.patients = 181, R = 39, NR = 133, NE = 9, NEs were not removed for survival analysis), **(j)** Javelin101 cohort (n.patients = 726), and **(k, l)** posttreatment CM009_POST cohort (n.patients = 55, R = 5, NR = 47, NE = 3) based on the Kaplan–Meier method. The groups were dichotomized at the median GSVA score for the CheckMate cohort and at the optimal cutoff point of the GSVA score for the other two bulk RNA-seq datasets, and the log-rank test was used to determine significance. Dashed line: median survival time. Color range: 95% CI. **(m)** The performance of univariate logistic regression model of MKI67$^+$Treg.Sig for predicting ICB outcomes.

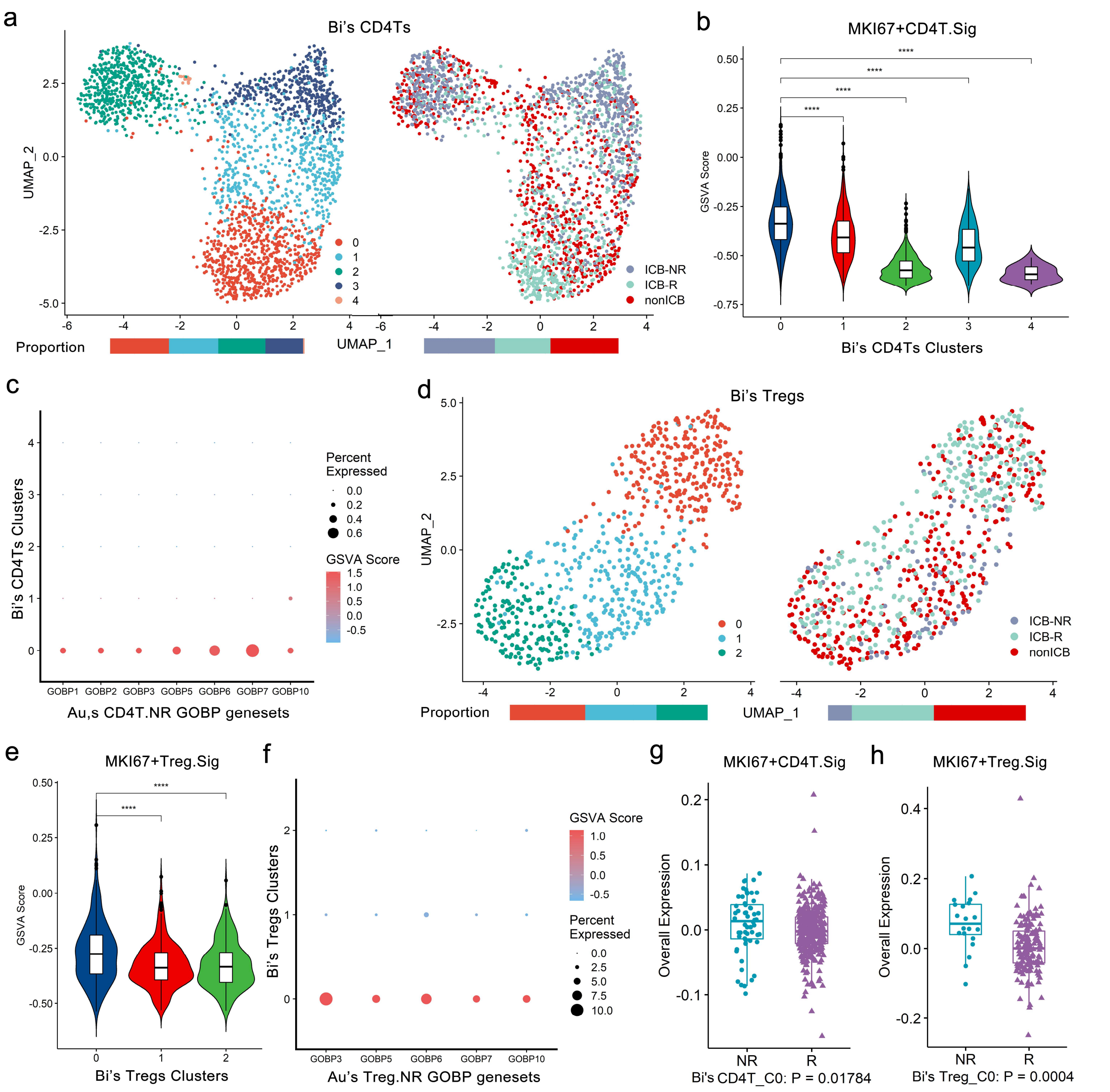

Supplementary Figure 6. Validation of the MKI67$^+$ CD4Ts and Treg signatures using an independent scRNA-seq dataset – Bi's dataset. (a) UMAP plot of CD4Ts in Bi's dataset that were classified into 5 subclusters from ICB-R, ICB-NR and NoICB samples. Bar plots show cell proportions grouped by clusters (left) and ICB outcomes (right). **(b)** Violin with boxplot plot of the GSVA score of MKI67$^+$CD4T.Sig in Bi's CD4Ts subclusters. Center line, median. Box limits, upper and lower quartiles. Whiskers, 1.5 interquartile range. Points beyond whiskers, outliers. A two-sided Wilcoxon test was used to determine significance. ****P < 0.0001. **(c)** Dot plot of the expression levels of 7 gene sets enriched in Au'sCD4T.NR with ROC adjusted p values < 0.05 in Bi's CD4Ts via GSVA analysis. Related to Fig. S2e and Extended data 2. **(d)** UMAP plot of Tregs in Bi's dataset that were classified into 3 subclusters from ICB-R, ICB-NR and NoICB samples. Bar plots show cell proportions grouped by clusters (left) and ICB outcomes (right). **(e)** Violin with boxplot plot of the GSVA score of MKI67$^+$Treg.Sig in Bi's Treg subclusters. Center line, median. Box limits, upper and lower quartiles. Whiskers, 1.5 interquartile range. Points beyond whiskers, outliers. A two-sided Wilcoxon test was used to determine significance. ****P < 0.0001. **(f)** Dot plot of the expression levels of 5 gene sets enriched in Au's Treg.NR with ROC adjusted p values < 0.05 in Bi's Tregs via GSVA analysis. Related to Fig. S2e and Extended data 2. **(g)** Boxplot validated that the Bi's CD4Ts_C0 subtype had significantly higher overall expression of MKI67$^+$CD4T.Sig in nonresponders than responders. Center line, median. Box limits, upper and lower quartiles. Whiskers, 1.5 interquartile range. Points beyond whiskers, outliers. A two-sided Wilcoxon test was used to determine significance. **(h)** Boxplot validated that Bi's Tregs_C0 subtype had significantly higher overall expression of MKI67$^+$Treg.Sig in non-responders than responders. Center line, median. Box limits, upper and lower quartiles. Whiskers, 1.5 interquartile range. Points beyond whiskers, outliers. A two-sided Wilcoxon test was used to determine significance.

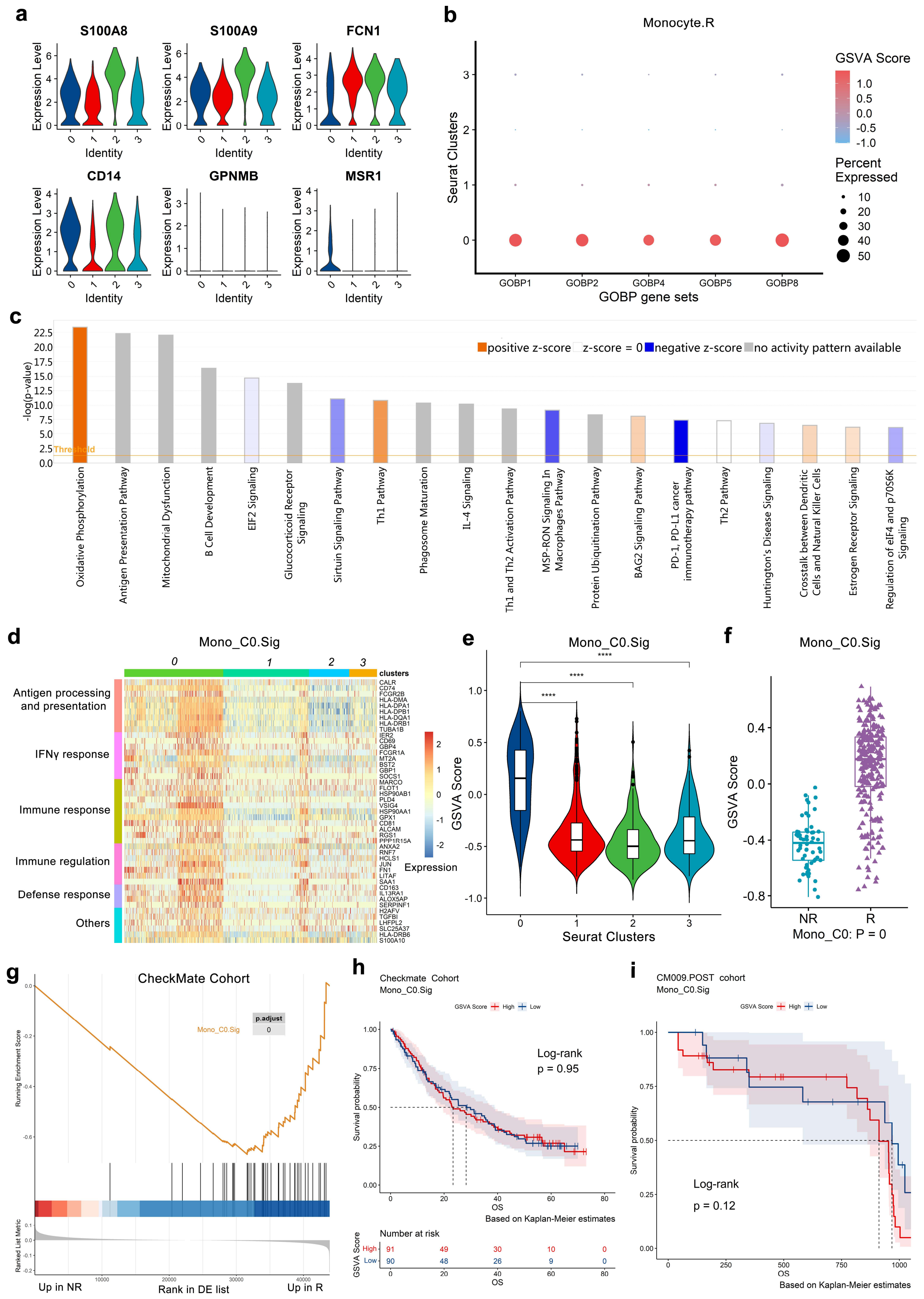
a
S100A8
S100A9
FCN1
CD14
GPNMB
MSR1
Expression Level
Identity
b
Monocyte.R
Seurat Clusters
GOBP1
GOBP2
GOBP4
GOBP5
GOBP8
GOBP gene sets
GSVA Score
Percent Expressed
c
-log(p-value)
positive z-score
z-score = 0
negative z-score
no activity pattern available
Threshold
Oxidative Phosphorylation
Antigen Presentation Pathway
Mitochondrial Dysfunction
B Cell Development
EIF2 Signaling
Glucocorticoid Receptor Signaling
Sirtuin Signaling Pathway
Th1 Pathway
Phagosome Maturation
IL-4 Signaling
Th1 and Th2 Activation Pathway
MSP-RON Signaling In Macrophages Pathway
Protein Ubiquitination Pathway
BAG2 Signaling Pathway
PD-1, PD-L1 cancer immunotherapy pathway
Th2 Pathway
Huntington's Disease Signaling
Crosstalk between Dendritic Cells and Natural Killer Cells
Estrogen Receptor Signaling
Regulation of eIF4 and p70S6K Signaling
d
Mono_C0.Sig
clusters
Antigen processing and presentation
IFNγ response
Immune response
Immune regulation
Defense response
Others
Expression
e
Mono_C0.Sig
GSVA Score
Seurat Clusters
f
Mono_C0.Sig
GSVA Score
NR
R
Mono_C0: P = 0
g
CheckMate Cohort
Running Enrichment Score
Mono_C0.Sig
p.adjust
Ranked List Metric
Up in NR
Rank in DE list
Up in R
h
Checkmate Cohort
Mono_C0.Sig
Survival probability
Log-rank
p = 0.95
OS
Based on Kaplan-Meier estimates
Number at risk
GSVA Score
High
Low
i
CM009.POST cohort
Mono_C0.Sig
Survival probability
Log-rank
p = 0.12
OS
Based on Kaplan-Meier estimates

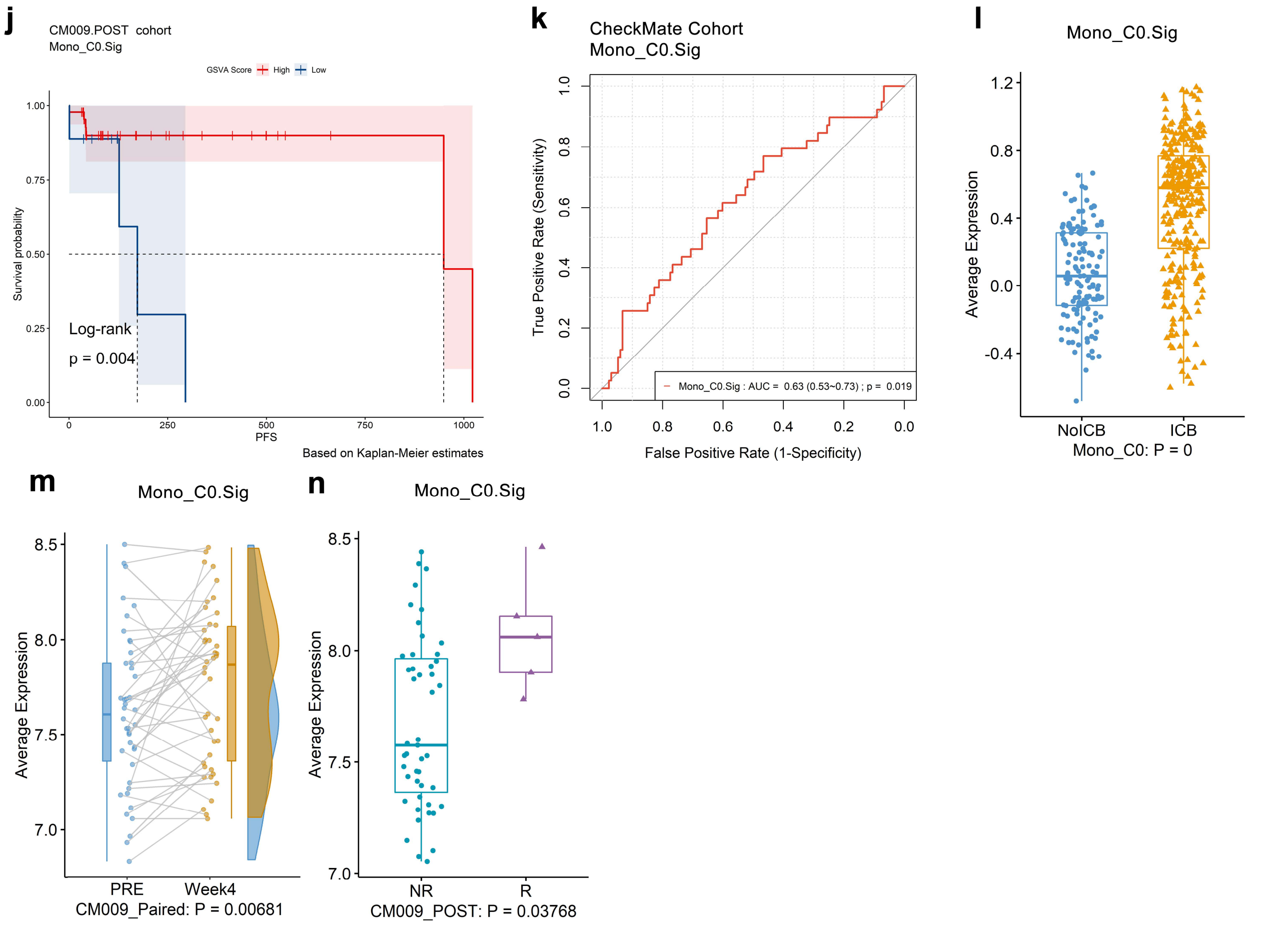

Supplementary Figure 7. A 45-gene signature penal that can specifically characterize the antigen presenting monocyte subtype and contribute to ICB response. The scRNA-seq dataset - Bi's monocytes and bulk RNA-seq datasets – CheckMate and CM009_POST – were analyzed. Related to Fig. 4. **(a)** Violin plot of the expression levels of classical monocyte marker genes in Bi's monocyte subclusters. S100A8, S100A9, FCN1 and CD14 are all monocyte markers; GPNMB and MSR1 are macrophage markers. **(b)** ICB response prediction monocyte subtype was identified by locating the effective predictive gene sets expression via GSVA method. Related to Fig. S2e and Extended data 2. **(c)** IPA results of Mono_C0 compared with other subclusters. The top 20 pathways sorted by the p-value are listed. Orange bars, activated; blue bars, suppressed. **(d)** Heatmap of the expression of a 45-gene signature penal (Mono_C0.Sig) representing the highly enriched pathways of antigen-presenting monocytes (Mono_C0). **(e)** Violin plot of Mono_C0.Sig GSVA scores showed that it can specifically characterize the Mono_C0 subtype. **(f)** Boxplot discovered that Mono_C0.Sig GSVA scores of R were significantly higher than those of NR in the Mono_C0 subtype via GSVA analysis. Center line, median. Box limits, upper and lower quartiles. Whiskers, 1.5 interquartile range. Points beyond whiskers, outliers. A two-sided Wilcoxon test was used to determine significance. **(g)** GSEA validated that Mono_C0.Sig was enriched in responders in the CheckMate cohort (n.patients = 172, R = 39, NR = 133). The p value was FDR-adjusted by the Benjamini–Hochberg method. **(h)-(j)** Survival analysis was performed on GSVA scores for Mono_C0.Sig in the pretreatment **(h)** CheckMate cohort (n.patients = 181, R = 39, NR = 133, NE = 9) and posttreatment **(i, j)** CM009_POST cohort based on the Kaplan–Meier method. The groups were dichotomized at the median GSVA score for the CheckMate cohort and at the optimal cutoff point of the GSVA score for the CM009_POST cohort (n.patients =55, R = 5, NR = 47, NE = 3), and the log-rank test was used to determine significance. Dashed line: median survival time. Color range: 95% CI. **(k)** The performance of univariate logistic regression model of Mono_C0.Sig for predicting ICB outcomes. **(l)** Boxplot discovered that the Mono_C0 subtype had significantly higher expression levels of Mono_C0.Sig in the ICB group compared with the NoICB group. Center line, median. Box limits, upper and lower quartiles. Whiskers, 1.5 interquartile range. Points beyond whiskers, outliers. A two-sided Wilcoxon test was used to determine significance. **(m)** Raincloudplot validated that the pretreatment and week 4 treatment paired samples' CM009 cohort (CM009_Paired, n.patients = 42) had significantly elevated expression levels of Mono_C0.Sig in week 4 treatment samples than pretreatment samples. Center line, median. Box limits, upper and lower quartiles. Whiskers, 1.5 interquartile range. Points beyond whiskers, outliers. Cloud, data distribution density. The paired Wilcoxon test was used to determine significance. **(n)** Boxplot validated that the posttreatment CM009_POST cohort (n.patients =52, R = 5, NR = 47) had significantly higher expression levels of Mono_C0.Sig in responders compared with nonresponders. Center line, median. Box limits, upper and lower quartiles. Whiskers, 1.5 interquartile range. Points beyond whiskers, outliers. A two-sided Wilcoxon test was used to determine significance.

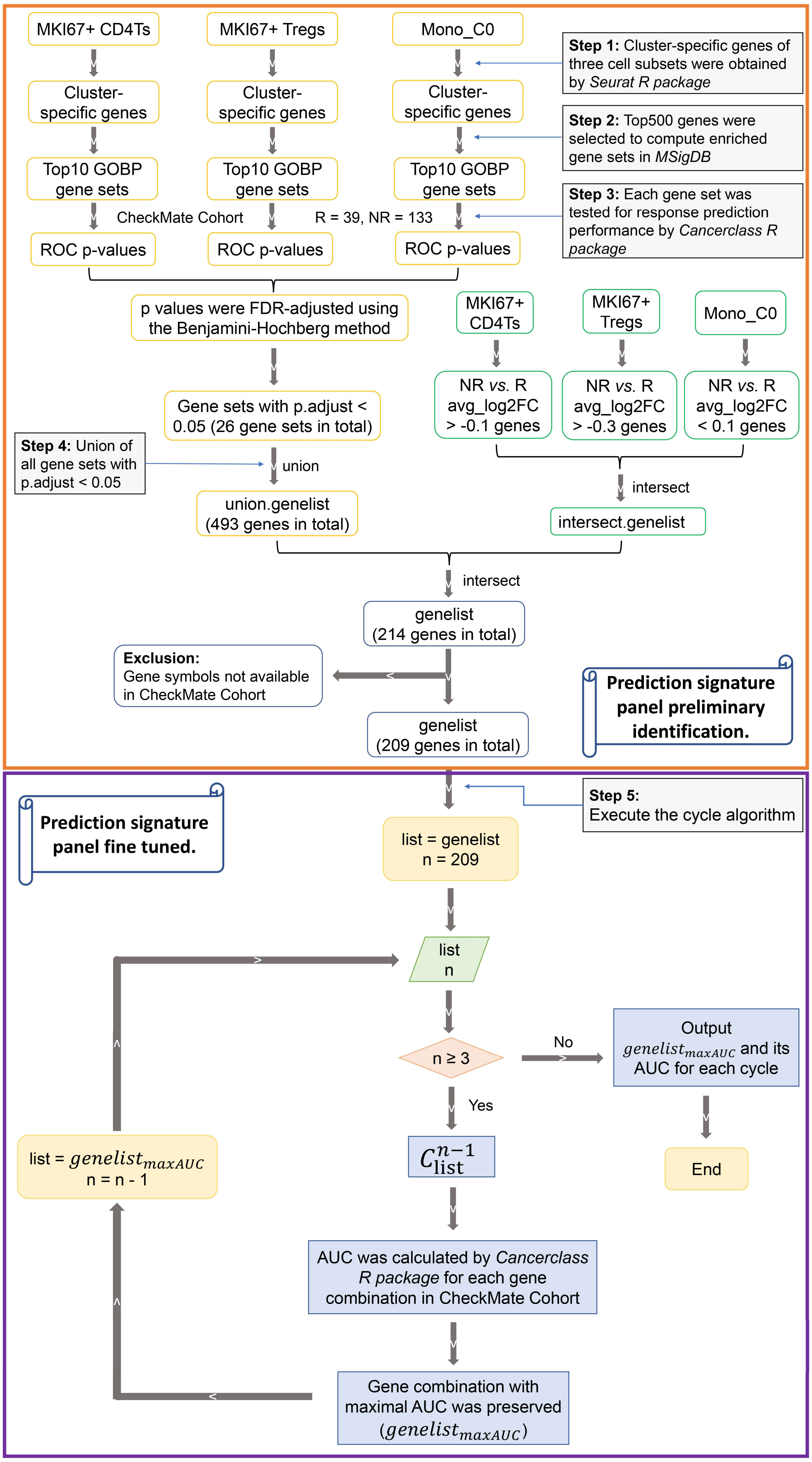

Supplementary Figure 8. Workflow of ICB response prediction signature construction.

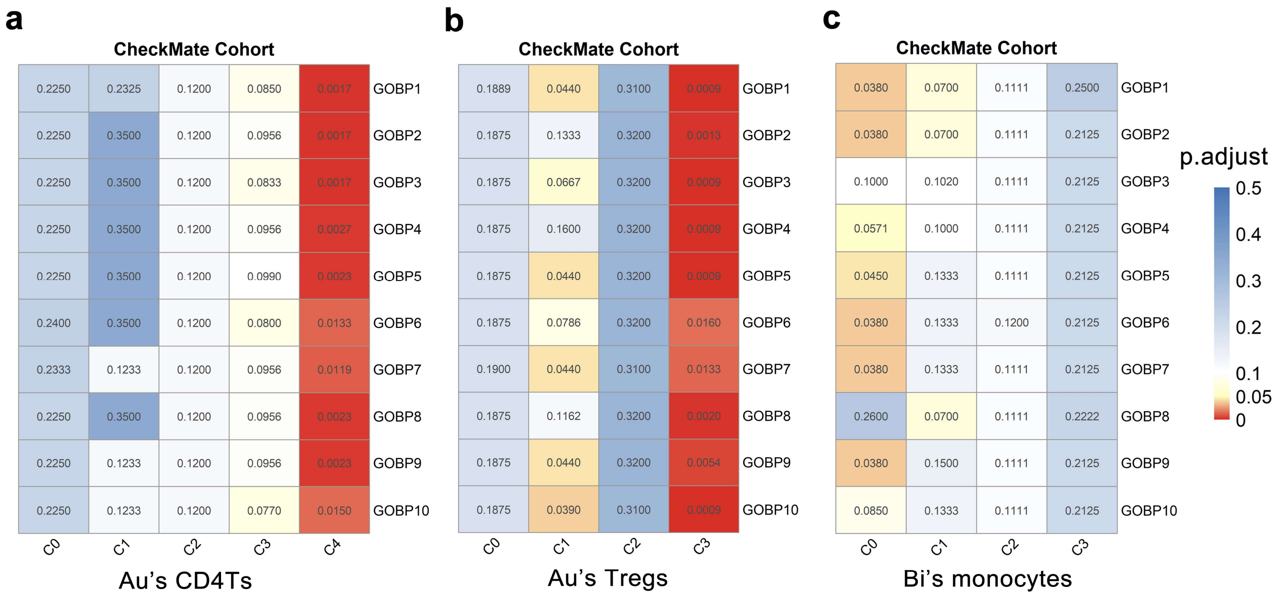

Supplementary Figure 9. Performances of 3 cell subtype cluster-specific marker gene-enriched gene sets for predicting ICB outcomes. Bi's and Au's datasets and CheckMate cohort (n.patients = 172, R = 39, NR = 133) were used in this analysis. All the pathways' details can be obtained from Extended data 5. Adjusted p values of the ROC curves for predicting ICB outcomes of enriched top 10 GOBP gene sets of **(a)** Au's CD4Ts, **(b)** Au's Tregs and **(c)** Bi's monocytes cluster-specific marker genes.

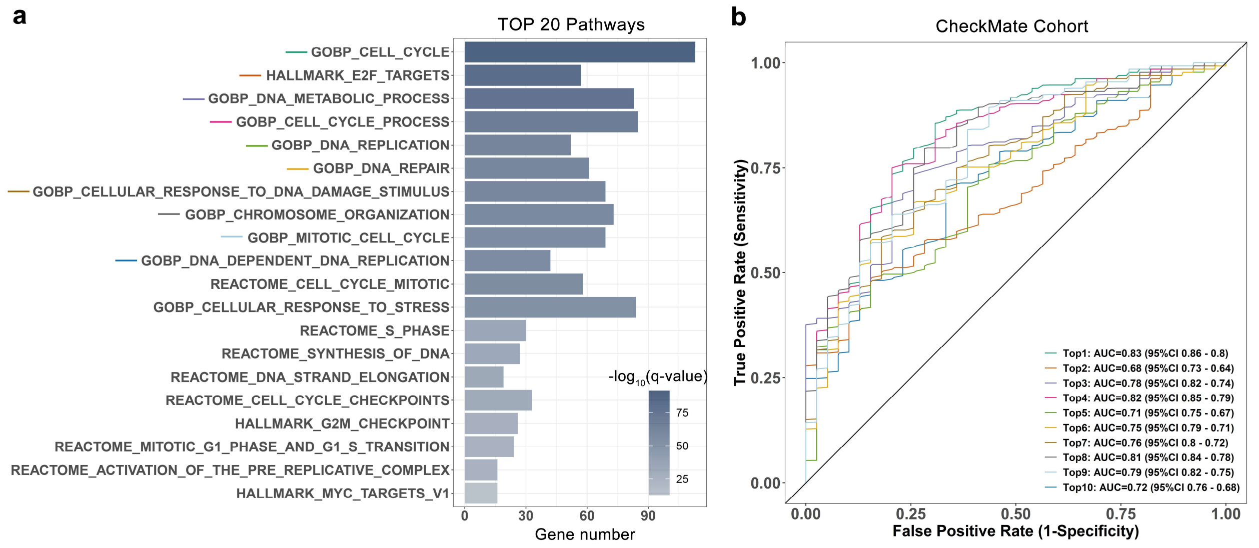

Supplementary Figure 10. Enrichment pathways of 209 genes and their predictive performance of ICB outcomes. CheckMate cohort (n.patients = 172, R = 39, NR = 133) was used in this analysis. **(a)** GOBP, Hallmark, KEGG and Reactome analysis results of 209 genes. The top 20 pathways sorted by the FDR-adjusted p-value (q-value) are listed. **(b)** The performances of the top 10 enrichment pathways in predicting ICB outcomes. See the left figure for these pathways' names.

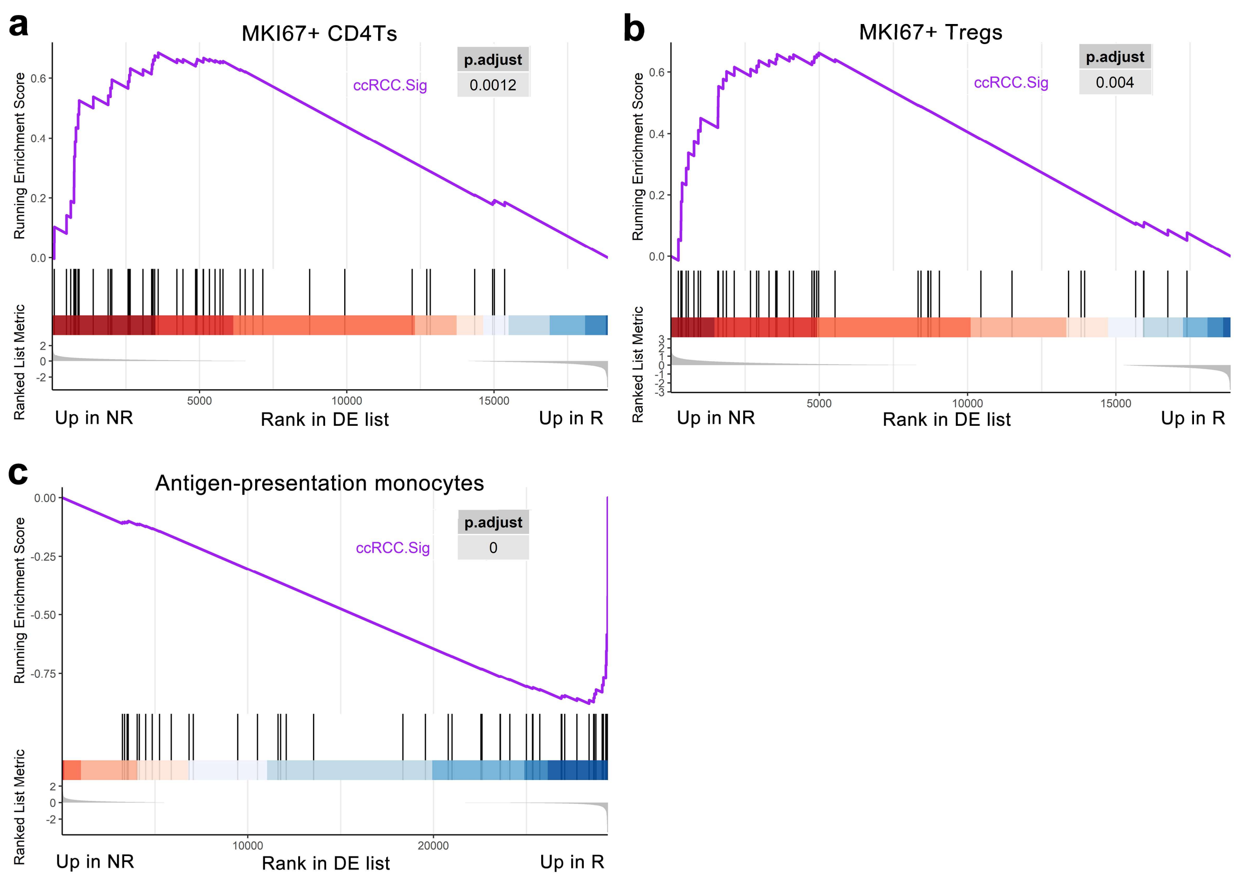

Supplementary Figure 11. The enrichment of ccRCC.Sig signature for the characteristic genes of 3 cell subtypes. Bi's dataset and Au's dataset were used in this analysis. ccRCC.Sig was significantly enriched in nonresponders of **(a)** MKI67^{+} CD4Ts and **(b)** MKI67^{+} Tregs and dramatically enriched in responders of **(c)** antigen-presenting monocytes. The p value was FDR-adjusted by the Benjamini–Hochberg method.

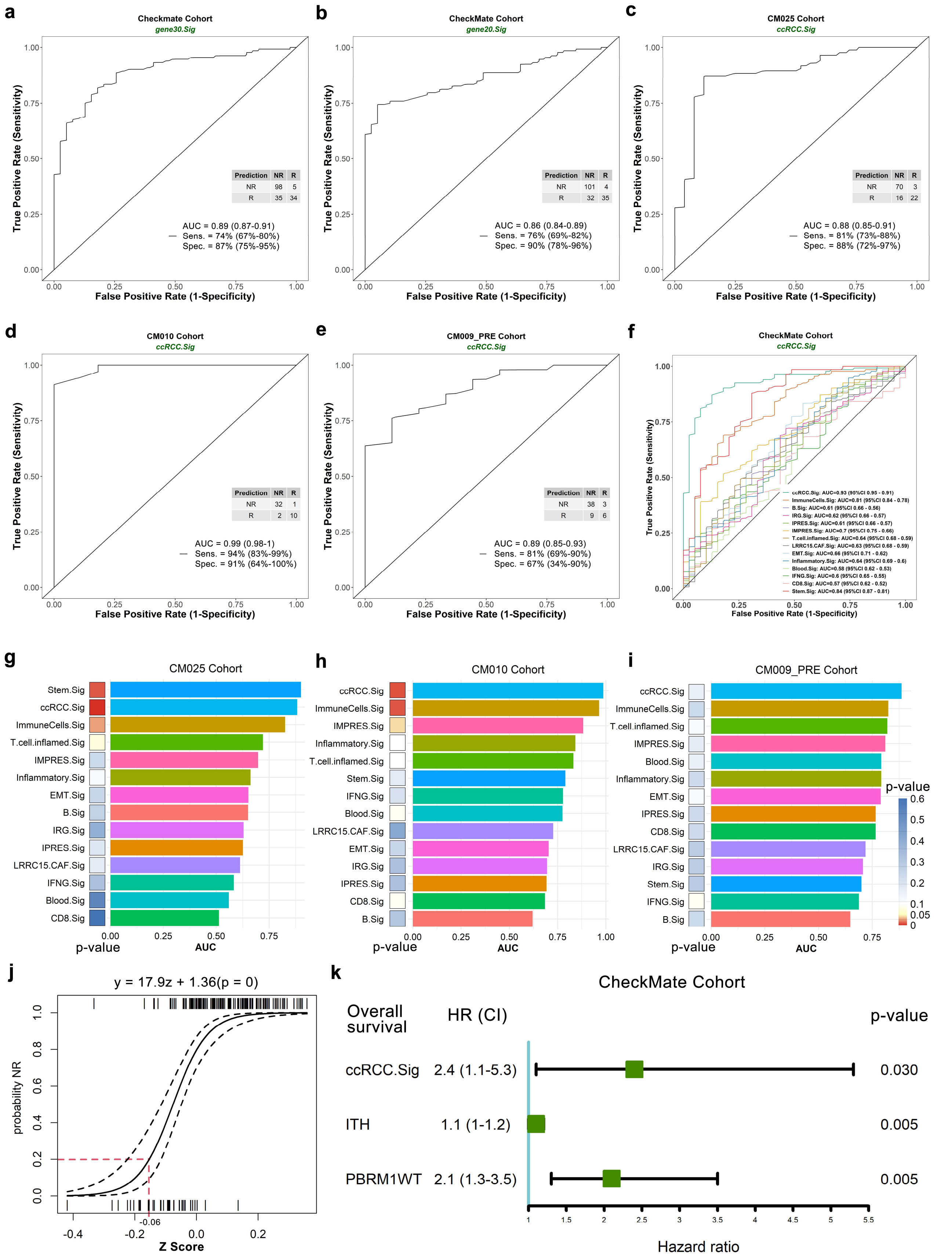

Supplementary Figure 12. Evaluation and comparison of the performance of ccRCC.sig. CheckMate (n = 172, R = 39, NR = 133), CM025 (n = 111, R = 25, NR = 86), CM010 (n = 45, R = 11, NR = 34) and CM009_PRE (n = 56, R = 9, NR = 47) cohorts were used in this analysis. Related to Fig. 5. **(a) and (b)** The performance of the **(a)** 30-gene combination and **(b)** 20-gene combination in the CheckMate cohort. **(c)-(e)** The performance of 47-gene ccRCC.Sig in the **(c)** CheckMate 025 (CM025), **(d)** CheckMate 010 (CM010) and **(e)** CheckMate 009 (CM009_PRE) cohorts. **(f)** Multiple ROC curves are shown for the 14 ICB response prediction signatures in the CheckMate cohort. **(g)-(i)** Comparison of the performance (AUC and p value) of ccRCC.Sig with 13 other ICB response signatures in the **(g)** CM025, **(h)** CM010 and **(i)** CM009_PRE cohorts. **(j)** ICB response prediction model, which can estimate the resistance probability (with 95% CI), constructed by converting continuous prediction scores (z score) of ccRCC.Sig in the CheckMate cohort through the logistic regression method embedded in the Cancerclass R package. **(k)** Multivariate Cox regression analyses of ccRCC.Sig and molecular features. ITH, intratumor heterogeneity. WT, wild type.

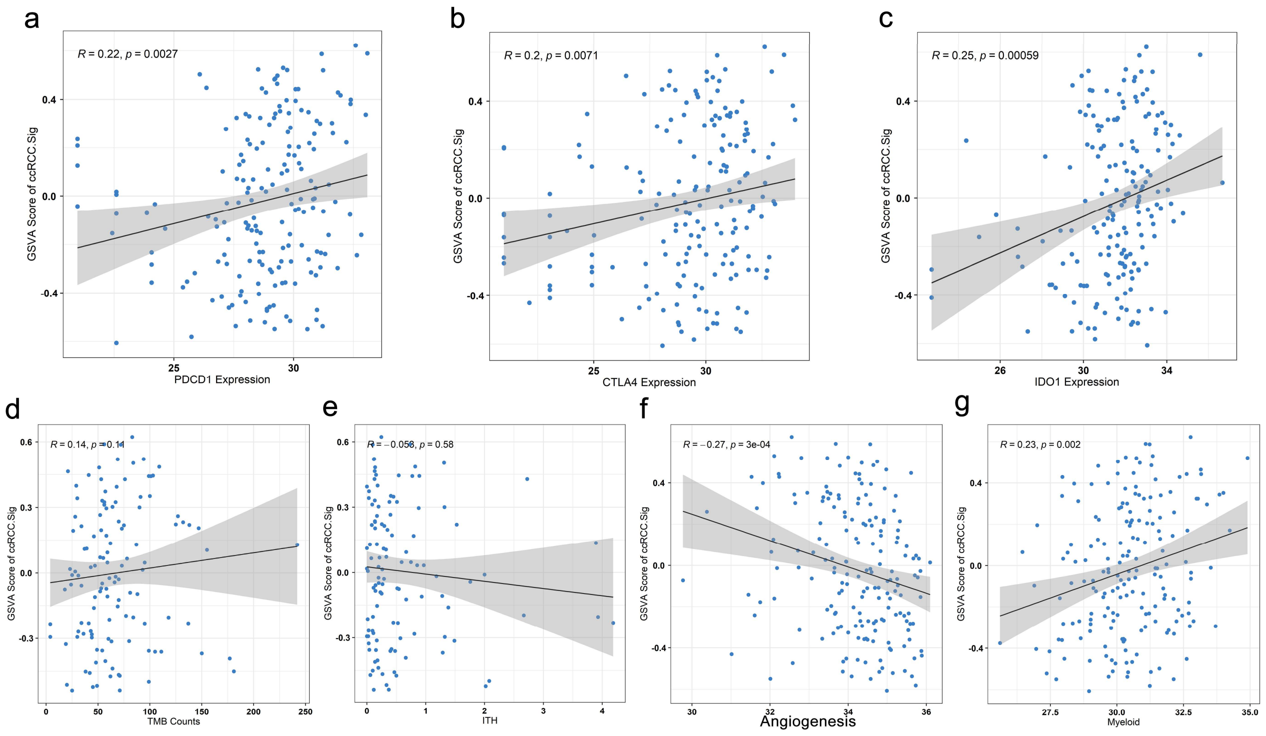

Supplementary Figure 13. Molecular correlation of ccRCC.Sig. CheckMate cohort was used in this analysis. Correlation of ccRCC.Sig and immune checkpoint molecules **(a-c)**, TBM counts **(d)**, ITH **(e)**, angiogenesis **(f)** and myeloid infiltration **(g)** in CheckMate cohort. GSVA scores were calculated to estimate the expression level of ccRCC.Sig for each sample.

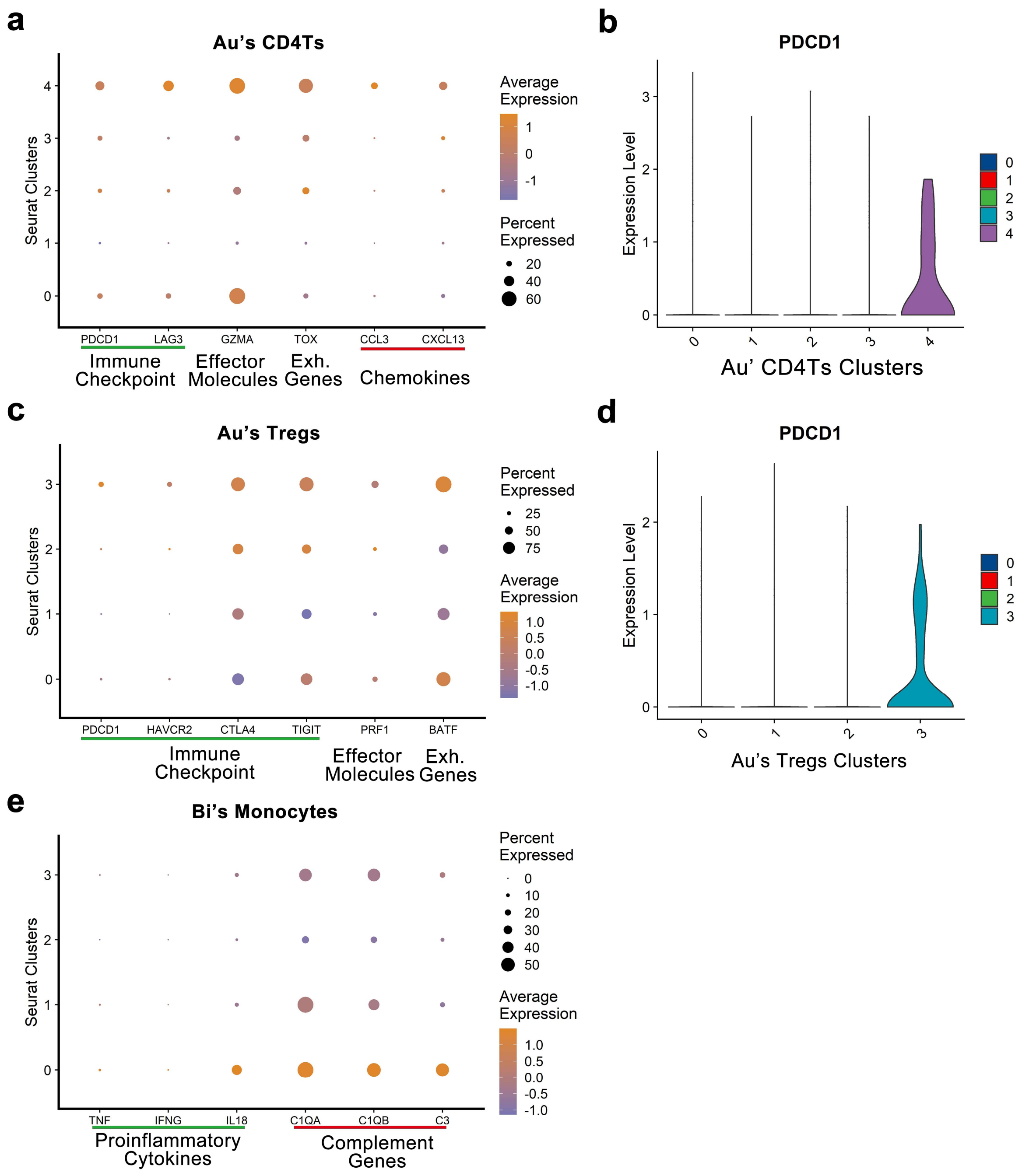

Supplementary Figure 14. Both MKI67$^+$ CD4Ts and Tregs are PD-1$^+$ proliferative populations and exhibit exhausted features. (a) Dot plot showing that immune checkpoint molecules, effector molecules, exhausted marker genes and chemokines were more highly expressed in MKI67$^+$ CD4Ts than in other subclusters. **(b)** Violin plot of the expression levels of PD-1 in Au's CD4T subclusters. **(c)** Dot plot showing that immune checkpoint molecules, effector molecules and exhausted marker genes were more highly expressed in MKI67$^+$ Tregs than in other subclusters. **(d)** Violin plot of the expression levels of PD-1 in Au Treg subclusters. **(e)** Dot plot showing the expression levels of proinflammatory cytokines and complement system genes in Bi's monocyte subcluster.

1.2 Supplementary Tables

Supplementary Table 1. Characteristics of ICB scRNA-seq datasets and bulk RNA-seq cohort.

Supplementary Table 2. Detailed information of ccRCC.Sig.

Supplementary Table 3. Thirteen published ICB response prediction signatures.

Supplementary Table 4. Comparison of AUC and p-value of previously published signatures.

1.3 Extended Data

Extended data 1. scCODE results (DE gene-lists) of responders and non-responders in 13 cell-types.

Extended data 2. Enriched GOBP, Hallmark, KEGG, and Reactome gene sets of 26 DE gene-lists and their ROC p-values.

Extended data 3. GSEA results of Bi's monocytes, Au's CD4Ts and Tregs.

Extended data 4. Details of MKI67+CD4T.Sig, MKI67+Treg.Sig and Mono_C0.Sig.

Extended data 5. Top10 GOBP gene sets of Bi's Monocytes, Au's CD4Ts and Tregs enriched by cluster specific marker-genes, and their ROC p-values.

Extended data 6. Gene combination with maximal AUC for each cycle (different gene-number combinations).
