# Supplementary material for: An immunotherapy response prediction model derived from proliferative CD4+ T cells and antigen-presenting monocytes in ccRCC
Source: Front Immunol. 2022 Aug 25;13:972227. doi: 10.3389/fimmu.2022.972227 (PMC9452905; doi:10.3389/fimmu.2022.972227)
Supplement: Supplementary file 2 [file DataSheet_2.pdf]

Supplementary Table 1. Characteristics of ICB scRNA-seq datasets and bulk RNA-seq cohort.

a. Single-cell RNA-seq datasets

Dataset Name	All Patients	Response			Treatment				Referances
		R (CR/PR)	NR (PD/SD)	NE	aPD-1	aPD-1+aCTLA-4	aPD1+TKI	NoICB	
**Bi's dataset**	8	2	2	1	2	1	2	3	(1)
**Au's dataset**	2	1	1	-	2 (Nivolumab)	-	-	-	(2)

b. Bulk RNA-seq cohorts

Cohorts	All Patients	Response			Treatment	OS	PFS	TMB	ITH	Sex	Age	PBRM1	VHL	Purity	Ploidy	References
		R (CR/PR)	NR (PD/SD)	NE												
**Checkmate**	181	39	133	9	Nivolumab	√	√	√	√	√	√	√	√	√	√	(3-6)
**Checkmate 025**	120	25	86	9	Nivolumab	√	√	√	√	√	√	√	√	√	√	(6)
**Checkmate 010**	45	11	34	-	Nivolumab	√	√	√	√	√	√	√	√	√	√	(5)
**Checkmate 009**																(4, 7)
CM009_PRE	59	9	47	3	Nivolumab	√	√	√	-	√	-	√	√	-	-	
CM009_POST	55	5	47	3	Nivolumab	√	√	√	-	√	-	√	√	-	-	
CM010_Paired	42	5	37	-	Nivolumab	√	√	√	-	√	-	√	√	-	-	
**PMC6035749**	17	5	12	-	Nivolumab	√	√	-	-	√	-	-	-	-	-	(8)
**GSE67501**	11	4	7	-	Nivolumab	√	√	-	-	√	√	-	-	-	-	(9)
**Javelin 101**	726	-	-	-	Avelumab+Axitinib: 354<br>Sunitinib: 372	-	√	-	-	√	√	-	-	-	-	(10)

Supplementary Table 2. Detailed information of ccRCC.Sig. Analysed by Ingenuity Pathway Analysis (IPA).

No.	Symbol	Entrez Gene Name	Location	Type(s)	Drug(s)
1	ALCAM	activated leukocyte cell adhesion molecule	Plasma Membrane	other	praluzatamab, ravtansine
2	ASF1B	anti-silencing function 1B histone chaperone	Nucleus	other	
3	C1QB	complement C1q B chain	Extracellular Space	other	
4	C1QC	complement C1q C chain	Extracellular Space	other	
5	C5AR1	complement C5a receptor 1	Plasma Membrane	G-protein coupled receptor	TJ210001, avacopan, IPH5401
6	CDC45	cell division cycle 45	Nucleus	other	
7	CDCA2	cell division cycle associated 2	Nucleus	other	
8	CENPQ	centromere protein Q	Nucleus	other	
9	CEP55	centrosomal protein 55	Cytoplasm	other	
10	CXCL10	C-X-C motif chemokine ligand 10	Extracellular Space	cytokine	MDX-1100
11	DNA2	DNA replication helicase/nuclease 2	Cytoplasm	enzyme	
12	DUT	deoxyuridine triphosphatase	Nucleus	enzyme	TAS-114
13	E2F2	E2F transcription factor 2	Nucleus	transcription regulator	
14	E2F3	E2F transcription factor 3	Nucleus	transcription regulator	
15	E2F8	E2F transcription factor 8	Nucleus	transcription regulator	
16	EGR1	early growth response 1	Nucleus	transcription regulator	
17	ESCO2	establishment of sister chromatid cohesion N-acetyltransferase 2	Nucleus	enzyme	
18	EXO1	exonuclease 1	Nucleus	enzyme	
19	FANCG	FA complementation group G	Nucleus	other	
20	FBXO43	F-box protein 43	Nucleus	other	
21	FCGR2B	Fc fragment of IgG receptor IIb	Plasma Membrane	transmembrane receptor	IgG, BI-1206, XmAb5871
22	FEN1	flap structure-specific endonuclease 1	Nucleus	enzyme	

23	HAVCR2	hepatitis A virus cellular receptor 2	Plasma Membrane	other	LY3415244, anti-TIM-3 monoclonal antibody, Sym023, RO7121661, TSR-022
24	HIRIP3	HIRA interacting protein 3	Nucleus	other	
25	HLA-DOA	major histocompatibility complex, class II, DO alpha	Plasma Membrane	transmembrane receptor	
26	HMGB3	high mobility group box 3	Nucleus	other	
27	IDO1	indoleamine 2,3-dioxygenase 1	Cytoplasm	enzyme	LY3381916, HTI-1090, navaximod, KHK2455, PF-06840003
28	IL18	interleukin 18	Extracellular Space	cytokine	
29	KIF14	kinesin family member 14	Cytoplasm	enzyme	
30	LIG1	DNA ligase 1	Nucleus	enzyme	
31	MBD3	methyl-CpG binding domain protein 3	Nucleus	other	
32	NEIL3	nei like DNA glycosylase 3	Nucleus	enzyme	
33	OIP5	Opa interacting protein 5	Nucleus	other	
34	PHPT1	phosphohistidine phosphatase 1	Cytoplasm	phosphatase	
35	POLA2	DNA polymerase alpha 2, accessory subunit	Nucleus	enzyme	
36	POLD1	DNA polymerase delta 1, catalytic subunit	Nucleus	enzyme	entecavir, cytarabine, clofarabine, tenofovir diphosphate, L-asparaginase
37	POLE	DNA polymerase epsilon, catalytic subunit	Nucleus	enzyme	cladribine, trifluridine, clofarabine, bortezomib
38	PTMS	parathymosin	Nucleus	other	
39	RAD51C	RAD51 paralog C	Nucleus	enzyme	
40	RBX1	ring-box 1	Cytoplasm	enzyme	
41	RMI2	RecQ mediated genome instability 2	Nucleus	other	
42	SPC24	SPC24 component of NDC80 kinetochore complex	Cytoplasm	other	
43	TRIP13	thyroid hormone receptor interactor 13	Cytoplasm	transcription regulator	
44	TTBK1	tau tubulin kinase 1	Other	kinase	
45	TUBA1C	tubulin alpha 1c	Cytoplasm	other	docetaxel, bevacizumab, cyclophosphamide, L-asparaginase,prednisone
46	VNN1	vanin 1	Plasma Membrane	enzyme	
47	XRCC2	X-ray repair cross complementing 2	Nucleus	enzyme	

Supplementary Table 3. Thirteen published ICB response prediction signatures.

Signature	References	Description
**B.Sig**	(11)	A 9-gene expression profiles of B cell markers.
**Stem.Sig**	(12)	A 454-gene stemness signature identified by large-scale single cell RNA sequencing analysis
**ImmuneCells.Sig**	(13)	A 108-gene expression signature of TREM2hi macrophages, γδ T cells and a subset of B cells.
**IFNG.Sig**	(14)	A 6-gene signature of interferon gamma (IFNγ) response biomarkers.
**CD8.Sig**	(15)	A 5-gene expression profiles of CD8+ T cell markers.
**IMPRES.Sig**	(16)	A 28 immune checkpoint genes signature that estimate immuno-predictive score (IMPRES).
**IRG.Sig**	(17)	A 11 immune-related genes (IRGs) signature for predicting cervical cancer patients' response to immune checkpoint inhibitors.
**LRRC15.CAF.Sig**	(18)	A 14-gene expression signature of LRRC15+ Cancer associated fibroblast.
**T.cell.inflamed.Sig**	(14, 19)	An 18-gene signature of T-cell–inflamed gene expression profiles that can predict clinical benefit of anti-PD-1 in pan-cancer.
**IPRES.Sig**	(20, 21)	A 16-gene signature involved in 73 pathways with innate anti-PD-1 resistance (IPRES).
**Inflammatory.Sig**	(21)	A 27 inflammation related genes expression signature that predicted response to immune checkpoint blockade in lung cancer.
**EMT.Sig**	(21)	A 12 epithelial-to-mesenchymal transition (EMT) related genes expression signature that predicted response to immune checkpoint blockade in lung cancer.
**Blood.Sig**	(22)	A 15-gene expression signature developed from blood samples that can predict response to anti-CTLA4 immunotherapy.

Supplementary Table 4. Comparison of AUC and p-value of previously published signatures.

Related to Figure 5 and Supplementary Figure 12.

a. AUC

Signatures	Checkmate	CM025	CM010	CM009_PRE	PMC6035749	GSE67501
ccRCC.Sig	0.93	0.88	0.99	0.89	0.87	1.00
B.Sig	0.61	0.65	0.62	0.65	0.73	0.46
Stem.Sig	0.84	0.90	0.79	0.70	0.91	0.92
ImmuneCells.Sig	0.81	0.83	0.97	0.83	1.00	1.00
IFNG.Sig	0.60	0.58	0.78	0.69	0.72	0.93
CD8.Sig	0.57	0.51	0.68	0.77	0.58	0.82
IMPRES.Sig	0.70	0.70	0.88	0.81	0.90	1.00
IRG.Sig	0.62	0.63	0.70	0.71	0.78	1.00
LRRC15.CAF.Sig	0.63	0.61	0.73	0.72	0.83	0.79
T.cell.inflamed.Sig	0.64	0.72	0.83	0.82	0.83	0.89
IPRES.Sig	0.61	0.63	0.69	0.77	0.97	0.82
Inflammatory.Sig	0.64	0.66	0.84	0.79	0.80	0.93
EMT.Sig	0.66	0.65	0.70	0.79	0.77	0.79
Blood.Sig	0.58	0.56	0.78	0.79	0.82	1.00

b. P-value

Signatures	Checkmate	CM025	CM010	CM009_PRE	PMC6035749	GSE67501
ccRCC.Sig	7.20E-07	0.00034	0.0084	0.16	0.29	0.11
B.Sig	0.28	0.27	0.31	0.24	0.81	0.59
Stem.Sig	0.0023	0.011	0.18	0.29	0.12	0.32
ImmuneCells.Sig	0.022	0.028	0.0096	0.24	0.16	0.11
IFNG.Sig	0.37	0.33	0.21	0.09	0.47	0.18
CD8.Sig	0.32	0.61	0.092	0.23	0.76	0.32
IMPRES.Sig	0.073	0.24	0.042	0.17	0.21	0.14
IRG.Sig	0.11	0.39	0.33	0.28	0.54	0.11
LRRC15.CAF.Sig	0.081	0.18	0.42	0.28	0.37	0.32
T.cell.inflamed.Sig	0.2	0.075	0.1	0.12	0.35	0.24
IPRES.Sig	0.21	0.19	0.33	0.23	0.33	0.39
Inflammatory.Sig	0.11	0.12	0.1	0.23	0.3	0.32
EMT.Sig	0.14	0.25	0.25	0.12	0.37	0.27
Blood.Sig	0.45	0.54	0.092	0.16	0.3	0.11

References

1. Bi K, He MX, Bakouny Z, Kanodia A, Napolitano S, Wu J, et al. Tumor and Immune Reprogramming During Immunotherapy in Advanced Renal Cell Carcinoma. *Cancer Cell* (2021) 39(5):649-61 e5. Epub 2021/03/13. doi: 10.1016/j.ccell.2021.02.015.
2. Au L, Hatipoglu E, Robert de Massy M, Litchfield K, Beattie G, Rowan A, et al. Determinants of Anti-Pd-1 Response and Resistance in Clear Cell Renal Cell Carcinoma. *Cancer Cell* (2021) 39(11):1497-518 e11. Epub 2021/10/30. doi: 10.1016/j.ccell.2021.10.001.
3. Braun DA, Hou Y, Bakouny Z, Ficial M, Sant' Angelo M, Forman J, et al. Interplay of Somatic Alterations and Immune Infiltration Modulates Response to Pd-1 Blockade in Advanced Clear Cell Renal Cell Carcinoma. *Nat Med* (2020) 26(6):909-18. Epub 2020/05/31. doi: 10.1038/s41591-020-0839-y.
4. Choueiri T, Fishman M, Escudier B, McDermott D, Drake C, Kluger H, et al. Immunomodulatory Activity of Nivolumab in Metastatic Renal Cell Carcinoma. *Clinical cancer research : an official journal of the American Association for Cancer Research* (2016) 22(22):5461-71. doi: 10.1158/1078-0432.Ccr-15-2839.
5. Motzer R, Rini B, McDermott D, Redman B, Kuzel T, Harrison M, et al. Nivolumab for Metastatic Renal Cell Carcinoma: Results of a Randomized Phase Ii Trial. *Journal of clinical oncology : official journal of the American Society of Clinical Oncology* (2015) 33(13):1430-7. doi: 10.1200/jco.2014.59.0703.
6. Motzer R, Tannir N, McDermott D, Arén Frontera O, Melichar B, Choueiri T, et al. Nivolumab Plus Ipilimumab Versus Sunitinib in Advanced Renal-Cell Carcinoma. *The New England journal of medicine* (2018) 378(14):1277-90. doi: 10.1056/NEJMoa1712126.
7. Mahoney KM, Ross-Macdonald P, Yuan L, Song L, Veras E, Wind-Rotolo M, et al. Soluble Pd-L1 as an Early Marker of Progressive Disease on Nivolumab. *J Immunother Cancer* (2022) 10(2). Epub 2022/02/09. doi: 10.1136/jitc-2021-003527.
8. Miao D, Margolis C, Gao W, Voss M, Li W, Martini D, et al. Genomic Correlates of Response to Immune Checkpoint Therapies in Clear Cell Renal Cell Carcinoma. *Science (New York, NY)* (2018) 359(6377):801-6. doi: 10.1126/science.aan5951.
9. Ascierto ML, McMiller TL, Berger AE, Danilova L, Anders RA, Netto GJ, et al. The Intratumoral Balance between Metabolic and Immunologic Gene Expression Is Associated with Anti-Pd-1 Response in Patients with Renal Cell Carcinoma. *Cancer Immunol Res* (2016) 4(9):726-33. Epub 2016/08/06. doi: 10.1158/2326-6066.CIR-16-0072.
10. Motzer RJ, Robbins PB, Powles T, Albiges L, Haanen JB, Larkin J, et al. Avelumab Plus Axitinib Versus Sunitinib in Advanced Renal Cell Carcinoma: Biomarker Analysis of the Phase 3 Javelin Renal 101 Trial. *Nat Med* (2020) 26(11):1733-41. Epub 2020/09/09. doi: 10.1038/s41591-020-1044-8.
11. Helmink BA, Reddy SM, Gao J, Zhang S, Basar R, Thakur R, et al. B Cells and Tertiary Lymphoid Structures Promote Immunotherapy Response. *Nature* (2020) 577(7791):549-55. Epub 2020/01/17. doi: 10.1038/s41586-019-1922-8.
12. Zhang Z, Wang Z, Chen Y, Wu H, Yin L, Zhao Q, et al. Integrated Analysis of Single-Cell and Bulk RNA Sequencing Data Reveals a Pan-Cancer Stemness Signature Predicting Immunotherapy Response. *Genome medicine* (2022) 14(1):45. doi: 10.1186/s13073-022-01050-w.
13. Xiong D, Wang Y, You M. A Gene Expression Signature of Trem2(Hi) Macrophages and Gammadelta T Cells Predicts Immunotherapy Response. *Nat Commun* (2020) 11(1):5084. Epub 2020/10/10. doi: 10.1038/s41467-020-18546-x.
14. Ayers M, Lunceford J, Nebozhyn M, Murphy E, Loboda A, Kaufman DR, et al. Ifn-Gamma-Related Mrna Profile Predicts Clinical Response to Pd-1 Blockade. *J Clin Invest* (2017) 127(8):2930-40. Epub 2017/06/27. doi: 10.1172/JCI91190.
15. Jiang P, Gu S, Pan D, Fu J, Sahu A, Hu X, et al. Signatures of T Cell Dysfunction and Exclusion Predict Cancer Immunotherapy Response. *Nat Med* (2018) 24(10):1550-8. Epub 2018/08/22. doi: 10.1038/s41591-018-0136-1.
16. Auslander N, Zhang G, Lee JS, Frederick DT, Miao B, Moll T, et al. Robust Prediction of Response to Immune Checkpoint

Blockade Therapy in Metastatic Melanoma. *Nat Med* (2018) 24(10):1545-9. Epub 2018/08/22. doi: 10.1038/s41591-018-0157-9.
17. Yang S, Wu Y, Deng Y, Zhou L, Yang P, Zheng Y, et al. Identification of a Prognostic Immune Signature for Cervical Cancer to Predict Survival and Response to Immune Checkpoint Inhibitors. *Oncoimmunology* (2019) 8(12):e1659094. Epub 2019/11/20. doi: 10.1080/2162402X.2019.1659094.
18. Dominguez CX, Muller S, Keerthivasan S, Koeppen H, Hung J, Gierke S, et al. Single-Cell RNA Sequencing Reveals Stromal Evolution into Lrrc15(+) Myofibroblasts as a Determinant of Patient Response to Cancer Immunotherapy. *Cancer Discov* (2020) 10(2):232-53. Epub 2019/11/09. doi: 10.1158/2159-8290.CD-19-0644.
19. Huang AC, Orlowski RJ, Xu X, Mick R, George SM, Yan PK, et al. A Single Dose of Neoadjuvant Pd-1 Blockade Predicts Clinical Outcomes in Resectable Melanoma. *Nat Med* (2019) 25(3):454-61. Epub 2019/02/26. doi: 10.1038/s41591-019-0357-y.
20. Hugo W, Zaretsky JM, Sun L, Song C, Moreno BH, Hu-Lieskovan S, et al. Genomic and Transcriptomic Features of Response to Anti-Pd-1 Therapy in Metastatic Melanoma. *Cell* (2016) 165(1):35-44. Epub 2016/03/22. doi: 10.1016/j.cell.2016.02.065.
21. Thompson JC, Hwang WT, Davis C, Deshpande C, Jeffries S, Rajpurohit Y, et al. Gene Signatures of Tumor Inflammation and Epithelial-to-Mesenchymal Transition (Emt) Predict Responses to Immune Checkpoint Blockade in Lung Cancer with High Accuracy. *Lung Cancer* (2020) 139:1-8. Epub 2019/11/05. doi: 10.1016/j.lungcan.2019.10.012.
22. Friedlander P, Wassmann K, Christenfeld AM, Fisher D, Kyi C, Kirkwood JM, et al. Whole-Blood RNA Transcript-Based Models Can Predict Clinical Response in Two Large Independent Clinical Studies of Patients with Advanced Melanoma Treated with the Checkpoint Inhibitor, Tremelimumab. *J Immunother Cancer* (2017) 5(1):67. Epub 2017/08/16. doi: 10.1186/s40425-017-0272-z.
